# A plant endophytic bacterium *Burkholderia seminalis* strain 869T2 increases plant growth under salt stress by affecting several phytohormone response pathways

**DOI:** 10.1186/s40529-025-00453-3

**Published:** 2025-02-04

**Authors:** Hau-Hsuan Hwang, Yu-Ting Huang, Pei-Ru Chien, Fan-Chen Huang, Chih-Lin Wu, Liang-Yu Chen, Shih-Hsun Walter Hung, I-Chun Pan, Chieh-Chen Huang

**Affiliations:** 1 Department of Life Sciences, National Chung Hsing University, No. 145, Xingda Road, Taichung, 402 Taiwan; 2 Department of Horticulture, National Chung Hsing University, No. 145, Xingda Road, Taichung, 402 Taiwan; 3 Innovation and Development Center of Sustainable Agriculture, National Chung Hsing University, Taichung, 402 Taiwan; 4 Advanced Plant and Food Crop Biotechnology Center, National Chung Hsing University, Taichung, 402 Taiwan; 5 Institute of Plant and Microbial Biology, Academia Sinica, Taipei, 115 Taiwan

**Keywords:** *Burkholderia*, Plant endophytic bacteria, Salt stress, Drought stress

## Abstract

**Background:**

Due to global warming and gradual climate change, plants are subjected to a wide range of environmental stresses, adversely affecting plant growth and production worldwide. Plants have developed various mechanisms to overpower these abiotic stresses, including salt stress, drought, and high light intensity. Apart from their own defense strategies, plants can get help from the beneficial endophytic bacteria inside host plants and assist them in enduring severe growth conditions. A previously isolated plant endophytic bacteria, *Burkholderia seminalis* 869T2, from vetiver grass can produce auxin, synthesize siderophore, and solubilize phosphate. The *B. seminalis* 869T2 can colonize inside host plants and increase the growth of bananas, *Arabidopsis*, and several leafy vegetables.

**Results:**

We further demonstrated that different growth parameters of *Arabidopsis* and pak choi plants were significantly increased after inoculating the *B. seminalis* 869T2 under normal, salt, and drought stress conditions compared to the mock-inoculated plants. Both transcriptome analysis and quantitative real-time PCR results showed that expression levels of genes related to phytohormone signal transduction pathways, including auxin, gibberellin, cytokinin, and abscisic acid were altered in *Arabidopsis* plants after inoculated with the strain 869T2 under salt stress, in comparison to the mock-inoculated control with salt treatments. Furthermore, the accumulation levels of hydrogen peroxide (H_2_O_2_), electrolyte leakage (EL), and malondialdehyde (MDA) were lower in the 869T2-inoculated *Arabidopsis* and pak choi plants than in control plants under salt and drought stresses.

**Conclusions:**

The plant endophytic bacterium strain *B. seminalis* 869T2 may affect various phytohormone responses and reduce oxidative stress damage to increase salt and drought stress tolerances of host plants.

**Supplementary Information:**

The online version contains supplementary material available at 10.1186/s40529-025-00453-3.

## Introduction

It has been estimated that 50% of yield loss of agricultural productivity is caused by abiotic stress globally (Krishnamoorthy et al. 2022; Watts et al. 2023). Abiotic stress contains all non-living components, including water, salinity, temperature, heavy metals, pH, and nutrient deficiency, all of which diminish crop productivity and viability. Soil salinity arises from natural causes like climate change and global warming, as well as poor water management, improper irrigation practices, and unsuitable fertilizer use (Malik and Arora 2022; Munns and Tester 2008; Van Zelm et al. 2020). High levels of soil salinity negatively affect plants in many different ways: decrease water and nutrient uptake from soils, reduce plant root and shoot growth, reductions of plant photosynthesis, chlorophyll content, mineral uptake, inhibitions of cell membrane and cell wall maturation, change in plant hormone balance, disturbance in protein metabolism, and productions of reactive oxygen species (ROS) (Ismail and Horie 2017; Munns and Gilliham 2015). Drought stress is another severe and emerging abiotic stress that is established when the humidity of the atmosphere and soil declines alongside rising air temperatures. Like salt stress, drought stress influences the leaf and soil water potential and transport of soil nutrients, reduces cell growth and division, and affects various physiological and biochemical processes, leading to overall decreased growth of agricultural crops (Li et al. 2009; Verma et al. 2021). Under various abiotic stresses, plants induce a series of reactions, including activation or inactivation of hormone-related signal transduction pathways, stress response genes, and functional enzymes to help plants tolerate stress (Munns and Tester 2008; Van Zelm et al. 2020). Besides their own sophisticated strategies to defend against abiotic stresses, plants can benefit from the organisms that live in close association with the plants. One group of these organisms are the endophytic bacteria that are beneficial, non-pathogenic, and utilize different mechanisms to strengthen plant growth and protect host plants from diverse abiotic and biotic stresses (Malik and Arora 2022; Peng et al. 2023; Verma et al. 2021). Further understanding of plant and endophytic bacteria interactions helps elucidate mechanisms for promoting plant stress tolerance and creating an eco-friendly and sustainable agriculture practice.

Endophytic bacteria are an endosymbiotic group of microorganisms that inhabit host plants and do not cause any symptomatic characteristics of disease. These bacteria can be found in most plant species and have been isolated from surface-sterilized plant tissues, such as roots, leaves, and stems, and a few from flowers, fruits, and seeds (Afzal et al. 2019; Lachu et al. 2022; Peng et al. 2023; Verma et al. 2021). The endophytic bacteria colonize the host plants and benefit from nutrients provided by the host plants. These bacteria usually initiate their colonization process from root wounds and breaks, followed by systemically colonizing aboveground parts of plants after their first establishment of a rhizospheric population in soils (Afzal et al. 2019; Santoyo et al. 2016). Most endophytic bacteria belong to the phylum Proteobacteria, Firmicutes, Actinobacteria, and Bacteroidetes (Santoyo et al. 2016; Peng et al. 2023). The two major isolated bacteria genera are *Bacillus* and *Pseudomonas*, and other commonly isolated genera are *Azoarcus*, *Burkholderia*, *Enterobacter*, *Herbaspirillum*, *Microbacterium*, *Micrococcus*, *Pantoea*, *Serratia*, *Streptomyces*, and *Stenotrophomonas* (Afzal et al. 2019; Peng et al. 2023). Various factors influence the assembly of endophytic bacteria communities, including environmental conditions, soil compositions, root exudates, various stages of plant development, types of analyzed plant tissues, and microbe-microbe and plant-microbe interactions (Peng et al. 2023).

Endophytic bacteria in plants play a vital role in protecting plants from a range of abiotic stresses. These bacteria can increase the more extended root hair numbers and help plants penetrate to the deeper layers of the soil and obtain more water under salt and drought stress (Malik and Arora 2022; Peng et al. 2023). Several endophytic bacteria utilize different mechanisms to directly mitigate stresses by increasing uptake of micronutrients from the soil that include nitrogen, phosphorous, zinc, or iron, via production of nitrogenase, solubilization of precipitated phosphates, excretion of chelating compounds, or expression of iron chelating agents siderophores in bacteria (Bhattacharjee et al. 2008; Olanrewaju et al. 2017; Palaniappan et al. 2010; Watts et al. 2023). The presence of endophytic bacteria often promotes plant growth and enhances tolerance to abiotic stresses because of the production or upregulation of various phytohormones, including auxin, cytokinin, gibberellins, ethylene, or abscisic acid (Gao et al. 2022; Verma et al. 2021; Watts et al. 2023). The auxin produced by bacteria can stimulate taproot/adventitious root growth that helps plants access more nutrients in soils (Peng et al. 2023). Auxin signaling is often linked to another stress-related phytohormone, ethylene, which increases salt tolerance by negatively affecting plant growth. Many endophytic bacteria secret the ACC deaminase enzyme, which breaks down ACC (an ethylene precursor) into beta-ketone glutaric acid and ammonia, thus reducing ethylene levels and relieving salt stress (Glick et al. 2007).

Other reports suggest that these endophytic bacteria help plants tolerate salt stress by changing the K^+^/Na^+^ ratio by the selective influx and efflux of sodium, potassium, and calcium along with an alter in the ratio of secondary metabolites and the contents of osmolytes (compatible solutes), such as proline, sugars (Gao et al. 2022; Watts et al. 2023). Abiotic stresses also induce ROS (Reactive Oxygen Species) productions that cause severe damage to cellular components, influence protein production and enzyme functions, change the redox status, peroxidize lipids, decrease membrane fluidity, and result in cell damage. The existence of endophytic bacteria induces enzymatic antioxidant systems, such as superoxide dismutase (SOD), catalase (CAT), ascorbate peroxidase (APX), and non-enzymatic antioxidants to decrease oxidative damage and alleviate abiotic stress in host plants (Ha-Tran et al. 2021; Singh et al. 2020).

A previously isolated endophytic bacterium *Burkholderia seminalis* strain 869T2 from surface-sterilized root tissues of vetiver grass (*Chrysopogon zizanioides*) showed plant growth promotion ability in banana, *Arabidopsis*, two heading leafy vegetables, cabbage and head lettuce, and several leafy vegetables, including ching chiang pak choi, pak choi, loose-leaf lettuce, romaine lettuce, red leaf lettuce, and Chinese amaranth (Ho et al. 2015; Hung et al. 2023; Hwang et al. 2021). The strain 869T2 had auxin production, siderophore synthesis, and phosphate solubilization abilities and helped banana plants decrease *Fusarium* wilt disease occurrences within bananas (Ho et al. 2015; Hwang et al. 2021). In addition, genomic sequences of the strain 869T2 revealed that it contained several genes related to molecular plant-endophyte interactions (MPEI), such as ACC deaminase and pyrroloquinoline quinone (PQQ) biosynthesis genes (Ho and Huang 2015; Hung et al. 2023). The strain 869T2 contained dioxin degradation-related genes and could degrade the toxic dioxin congener 2,3,7,8-tetrachlorinated dibenzo-p-dioxin (TCDD) mainly via its 2-haloacid dehalogenase (2-HAD) (Ho and Huang 2015; Nguyen et al. 2021).

Several studies have shown that other *Burkholderia seminalis* strains enhance plant growth and increase plant pathogen resistance (Parke and Gurian-Sherman 2001). The *B. seminalis* TC3.4.2R3 strain isolated from sugarcane can decrease infections of *Fusarium oxysporum*, the cacao pathogens *Moniliophthora perniciosa* (fungus), *Phytophthora citrophtora*, *P. capsici*, and *P. palmivora* (oomycete), and the orchid necrosis by *Burkholderia gladioli* via producing a rhamnolipid and other diffusible metabolites (Araújo et al. 2016, 2017a, b; Luvizotto et al. 2010). The rice rhizosphere soil-located *B. seminalis* R456 strain showed decreased infection of the rice sheath blight (ShB) disease caused by *Rhizoctonia solani* (Li et al. 2011; Zhang et al. 2020). Additionally, a *B. seminalis* strain isolated from India can produce auxin, indole acetic acid (IAA), and increase tomato seedling growth (Tallapragada et al. 2015). Another *B. seminalis* ASB21 strain can produce auxin and enhance rice seedling growth (Panhwar et al. 2015). Similar plant growth promotion ability was observed in the *B. phytofirmans* PsJN strain, isolated from onion roots, that boosted the growth of potato, tomato, *Arabidopsis*, grapevines, switch-grass, maize, and wheat (Naveed et al. 2014, 2015; Pillay and Nowak 1997; Sessitsch et al. 2005; Sheibani-Tezerji et al. 2015).

In addition to the plant growth promotion ability of the *B. seminalis* strain 869T2, we have further demonstrated that inoculations of the strain 869T2 in *Arabidopsis*, pak choi, and Chinese amaranth plants can increase host plant tolerance to salt and drought stresses in this study. To further understand how strain 869T2 helps plants increase abiotic stress tolerances, the transcriptome analysis, quantitative real-time PCR, and physiological analysis results were subsequently performed. These analysis results showed that strain 869T2 may alleviate stress impairment of host plants by influencing various phytohormone responses and decreasing oxidative stress damage inside plant cells.

## Results

### Inoculation of *B. Seminalis* strain 869T2 into *Arabidopsis* plants improved plant growth under the salt stress

**Fig. 1:**
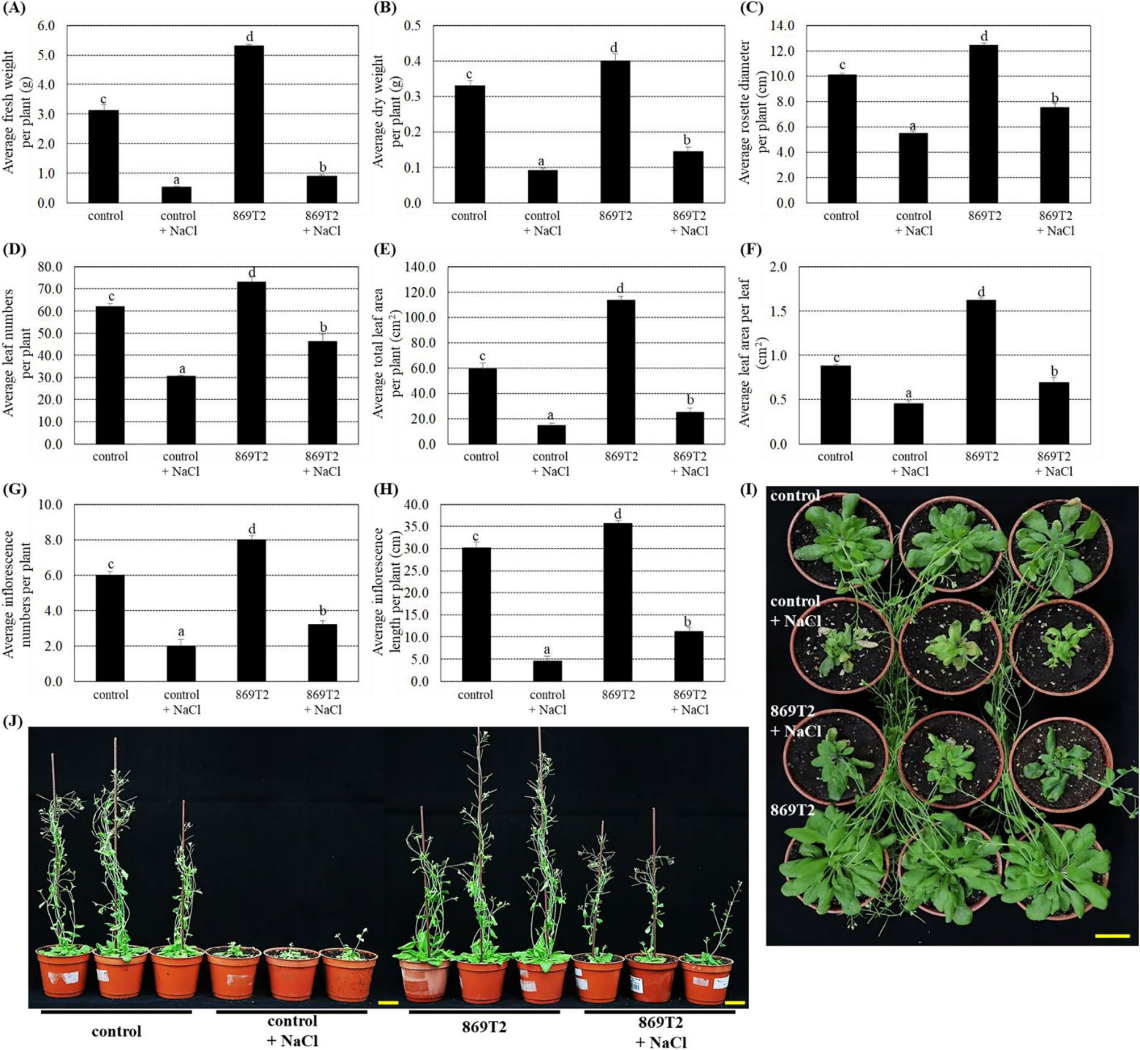
Under salt stress treatments, inoculations of the strain 869T2 in the *Arabidopsis* enhanced its growth compared to the mock-inoculated control plants. After 869T2 inoculations, the plants were then treated with the 250 mM NaCl solutions for five days to create salt stress treatments and subsequently recovered for three days by re-watering with distilled water. After salt stress treatments, the average fresh weight per plant (Panel **A**), the average dry weight per plant (Panel **B**), the average rosette diameter per plant (Panel **C**), the average leaf numbers per plant (Panel **D**), the average total leaf area per plant (Panel **E**), the average leaf area per leaf (Panel **F**), the average inflorescence length per plant (Panel **G**), the average inflorescence numbers per plant (Panel **H**) of the control and the 869T2-inoculated plants under salt stress and non-stress conditions were recorded. Data are mean±SE (standard error) from at least three independent bacteria inoculation experiments. More than 20 individual plants were examined for each bacteria inoculation assay. Data were analyzed by Duncan tests and means with different letters were significantly different (*p* < 0.05). The top-view (Panel **I**) and side-view (Panel **J**) photographs of the mock-inoculated control and the 869T2-inoculated plants after salt stress treatments. Yellow bar = 3 cm

Previous studies by Hung et al. (2023) and Hwang et al. (2022) demonstrated that the *B. seminalis* strain 869T2 can successfully colonize plants and increase the growth of *Arabidopsis* and several leafy vegetables. Data in Fig. 1 demonstrated that inoculations of the strain 869T2 significantly increased the average *Arabidopsis* plant fresh weight (Fig. 1A), dry weight (Fig. 1B), rosette diameter (Fig. 1C), leaf numbers (Fig. 1D), total leaf area per plant (Fig. 1E), leaf area per leaf (Fig. 1F), inflorescence numbers (Fig. 1G), and inflorescence length (Fig. 1H) comparing with mock-inoculated controls, which is consistent with previous studies.

We further examined if the strain 869T2 could affect plant growth under abiotic stress conditions. The mock-inoculated control and 869T2-inoculated plants were treated with 250 mM NaCl for five days and allowed the plants to recover for three days by watering with distilled water. After salt stress treatments, the control and 869T2-inoculated plants showed significant reductions in different plant growth parameters (Fig. 1), indicating that salt stress substantially affected plant growth. However, when grown under salt stress, the 869T2-inoculated plants had more than a 1.4-fold increase in various plant growth parameters compared with mock-inoculated control plants (Fig. 1). Figure 1I and J also showed that 869T2-inoculated plants were larger and had more leaves than control plants. This suggests the inoculation of *Arabidopsis* with the strain 869T2 promoted plant growth under normal growth and salt stress conditions.

### Differential transcriptional responses of the 869T2-inoculated *Arabidopsis* to salt stress

**Fig. 2:**
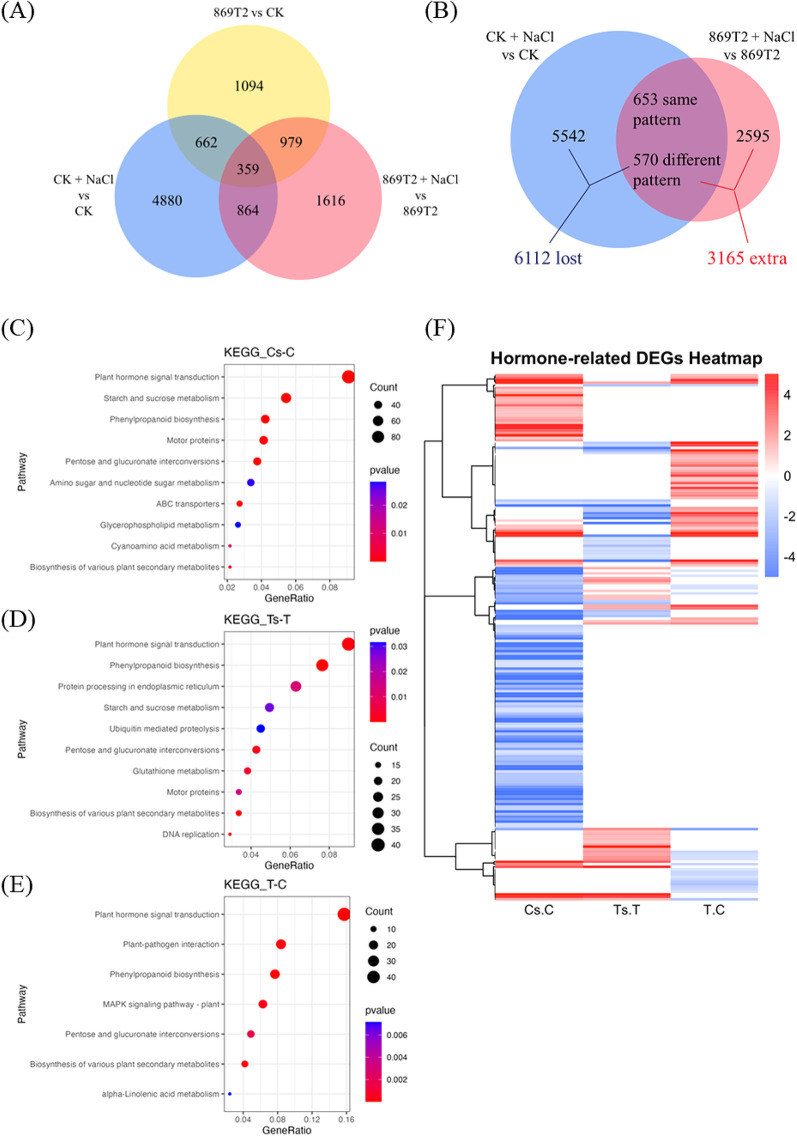
Transcriptome analysis of salt stress treated and 869T2-inoculated *Arabidopsis* plants. The Venn diagram of DEGs in different comparative groups (Panel **A**) and DEGs from salt stress-treated vs. untreated control (C_NaCl vs. C) and salt stress-treated and 869T2-inoculated vs. 869T2-inoculated only (T_NaCl vs. T) (Panel **B**). The enriched KEGG pathways of DEGs in C_NaCl (Cs) vs. C, T_NaCl (Ts) vs. T, and 869T2-inoculated vs. mock-inoculated control (T vs. C) (Panel **C**, **D**, **E**). The heatmap of significantly regulated DEGs related to plant hormones signal transduction pathways (Panel **F**). The columns represent the expression levels of DEGs based on log2 normalized read counts. The T represented the 869T2 inoculated plant sample. The Ts represented the salt stress-treated and 869T2-inoculated plant sample. The C represented the mock-inoculated control plant sample. The Cs represented the salt stress-treated control plant sample

To understand the possible mechanism of how the *B. seminalis* strain 869T2 promotes plant growth and salt tolerance, the transcriptomic response to salt stress in *Arabidopsis* seedlings inoculated with the strain 869T2 was further investigated. The RNA-Seq analysis was conducted on 869T2-inoculated and mock-inoculated control plants exposed to salt treatments. A total of 10,454 differentially expressed genes (DEGs) were identified and categorized into three comparative groups for analysis: 869T2-inoculated vs. mock-inoculated control (T vs. C), salt stress-treated vs. untreated control (C_NaCl vs. C), and salt stress-treated and 869T2-inoculated vs. 869T2-inoculated only (T_NaCl vs. T). Venn analysis revealed distinct patterns of salt stress-responsive DEGs, particularly in plants inoculated with 869T2 (Fig. 2A). Specifically, 4,880, 1,616, and 1,094 DEGs were uniquely expressed in C_NaCl vs. C, T_NaCl vs. T, and T vs. C, respectively. Over 80% of DEGs in C_NaCl vs. C differed from those in T_NaCl vs. T (Fig. 2B). Among these, 6,112 DEGs in C_NaCl vs. C exhibited no differential expression or underwent a reversal in the regulation direction in T_NaCl vs. T. Additionally, 3,165 extra DEGs were observed in T_NaCl vs. T compared to C_NaCl vs. C. These findings suggest that the expression of genes involved in salt stress response is altered following inoculation with 869T2.

The KEGG analysis was performed to investigate further the functional roles of DEGs influenced by inoculation with the 869T2 under salt stress conditions. The results of the KEGG-based DEG enrichment revealed notable findings. The category exhibiting the highest enrichment was “plant hormone signal transduction,” with 97 DEGs in C_NaCl (Cs) vs. C, 40 DEGs in T_NaCl (Ts) vs. T, and 45 DEGs in 869T2-inoculated vs. mock-inoculated control (T vs. C) (Fig. 2C, D and E). Additionally, common categories displaying significant enrichment included “phenylpropanoid biosynthesis” across all three comparative groups, and “starch and sucrose metabolism” is high enrichment in C_NaCl (Cs) vs. C and T_NaCl (Ts) vs. T (Fig. 2C, D and E). These findings contribute valuable insights toward comprehending the complicated molecular mechanisms underlying the salt stress response influenced by strain 869T2.

We observed differential expression of genes related to hormone signal transduction pathways, encompassing auxin, gibberellin, cytokinin, ethylene, and abscisic acid (ABA) when comparing control plants with those inoculated with 869T2 under salt stress conditions (Fig. 2F). Many down-regulated DEGs observed in control plants under salt stress exhibited either no differential expression or a reversal in the regulation direction in 869T2-inoculated plants under salt stress. Together with our previous findings demonstrating enhanced salt-stress tolerance in 869T2-inoculated plants, these results suggest that 869T2 may modulate plant response to salt stress through systemic regulation of phytohormone signaling pathways in general.

To validate our RNA-Seq results, we performed quantitative real-time PCR **(**qPCR) analysis on *Arabidopsis* plants subjected to different treatments: the mock-inoculated control, control with salt treatments (+ NaCl), the 869T2-inoculated, and the 869T2 inoculated with salt treatments. Like RNA-Seq results, the qPCR analysis showed that the 869T2 inoculation in plants significantly up-regulated transcript levels of several genes associated with both growth enhancement and stress-responsive hormones.

**Fig. 3:**
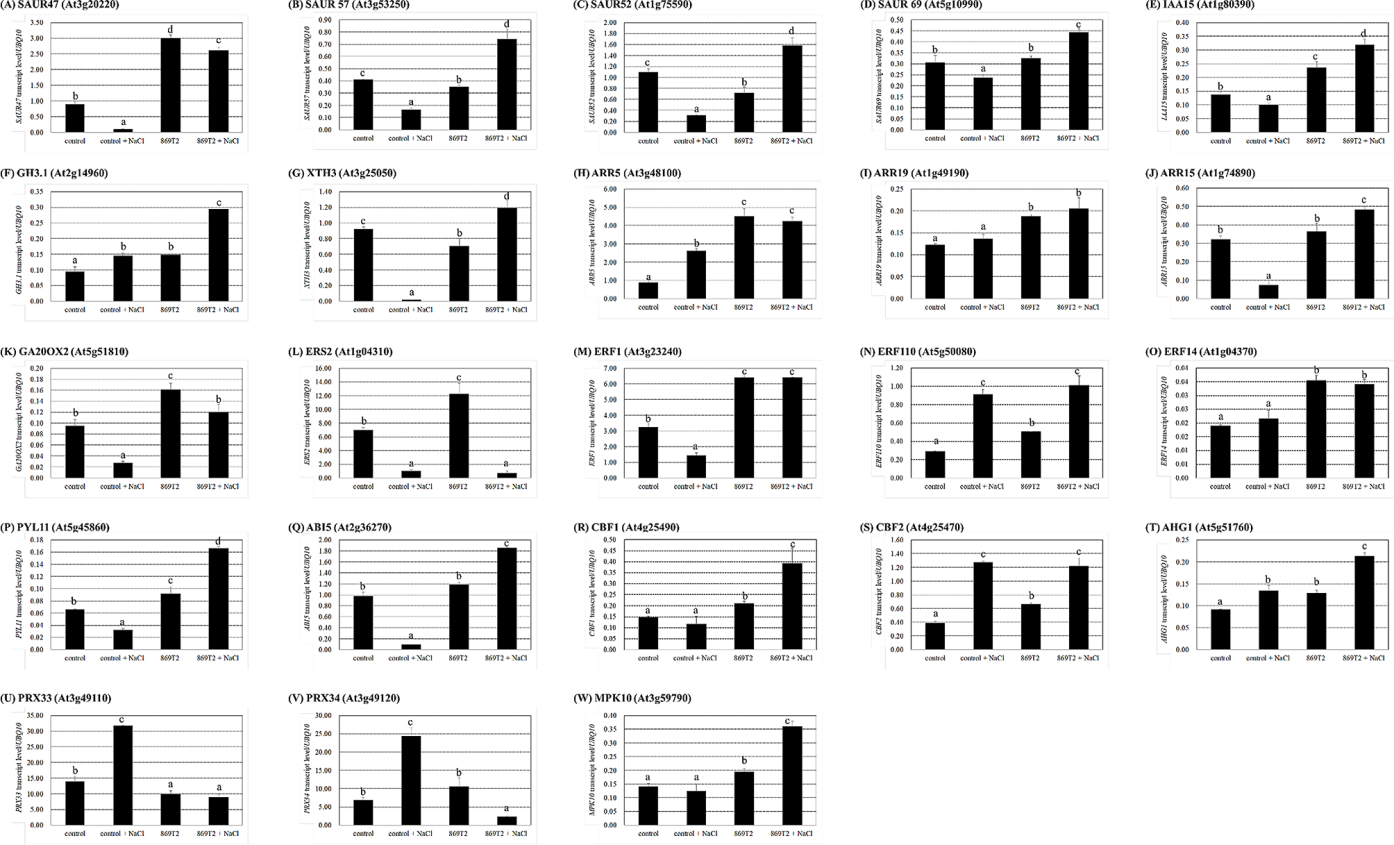
The transcript levels of auxin, cytokinin, GA, ethylene, and ABA response-related genes differed in the mock-inoculated control and the 869T2-inoculated *Arabidopsis* plants under non-stress and salt stress conditions. Transcript levels of the auxin-responsive genes: *SAUR47* (Panel **A**), *SAUR57* (Panel **B**), *SAUR52* (Panel **C**), *SAUR69* (Panel **D**), *IAA15* (Panel **E**), *GH3.1* (Panel **F**), *XTH3* (Panel **G**), the cytokinin response regulators: *ARR5* (Panel **H**), *ARR19* (Panel **I**), *ARR15* (Panel **J**), the GA biosynthesis gene: *GA20OX2* (Panel **K**), the ethylene-related genes: *ERS2* (Panel **L**), *ERF1* (Panel **M**), *ERF110* (Panel **N**), *ERF14* (Panel **O**), the ABA-responsive genes: *PYL11* (Panel **P**), *ABI5* (Panel **Q**), *CBF1* (Panel **R**), *CBF2* (Panel **S**), *AHG1* (Panel **T**), the ROS-responsive genes: *PRX33* (Panel **U**), *PRX34* (Panel **V**), and the MAPK signaling gene: *MPK10* (Panel **W**) in control and 869T2-inoculated *Arabidopsis* plants with and without salt treatments were measured by qPCR analysis. The *UBQ10* (polyubiquitin 10) transcript level was an internal control. Data are mean±SE from at least three independent biological experiments. Data were analyzed by Duncan test, and different letters were significantly different (*p* < 0.05)

Under salt stress, several auxin-responsive gene expressions, including *SMALL AUXIN UP RNA (SAUR)47*,* SAUR57*,* SAUR52*,* SAUR69*,* INDOLE-3-ACETIC ACID (IAA)15*, and xyloglucan endotransglucosylase/hydrolase *(XTH) 3* were repressed (Fig. 3A-E and G). On the contrary, the transcript levels of those auxin-related genes,, *SAUR47*,* SAUR57*,* SAUR52*,* SAUR69*,* IAA15*,* GH3.1*, and *XTH3*, were significantly up-regulated after the bacteria 869T2 and salt treatments (Fig. 3A-G) compared to the control with salt treatments. In addition, the cytokinin response regulators, *ARR5*,* ARR19*, and *ARR15*, as well as the gibberellin biosynthesis gene *GIBBERELLIN 20 OXIDASE 2* (*GA20OX2*), exhibited increased transcript levels in the 869T2-inoculated with salt stress treated plants compared to the mock-inoculated control with salt treatments (Fig. 3H-K). These data suggested the enhanced responses in the auxin and cytokinin signaling, as well as gibberellin synthesis with the strain 869T2 inoculation in *Arabidopsis* plants after salt treatments.

For stress-responsive hormones, the ethylene signaling response-related genes, including the *ETHYLENE RESPONSE SENSOR2* (*ERS2*), *ETHYLENE RESPONSE FACTOR 1* (*ERF1*), *ERF110*, and *ERF14*, were significantly induced in the 869T2-treated plants compared to the control (Fig. 3L-O). Furthermore, the *ERF1* and *ERF14* transcript levels were relatively higher in the 869T2 with NaCl plant sample compared to the control with NaCl sample (Fig. 3M and O). The ABA signaling response-related genes, the *PYRABACTIN RESISTANCE-LIKE 11 (PYL11)*,* C-Repeat Binding Factor 1 (CBF1)*,* CBF2*, and *ABA hypersensitive germination 1 (AHG1)* genes were up-regulated in the 869T2-treated plants compared to the control (Fig. 3P, R-T). Additionally, the *PYL11*, *ABA-insensitive 5 (ABI5)*, *CBF1*, and *AHG1* genes showed higher expression levels in the 869T2 with NaCl sample than the control with NaCl sample (Fig. 3P-R and T). These data suggested that the ethylene and ABA signaling responses were hyper-stimulated in the 869T2-treated plants under salt stress.

Under salt stress, the *PRX33* and *PRX34* genes, encoding the class III peroxidases and can contribute ROS productions, augmented significantly in the control plants, whereas these gene expression levels considerably declined after the strain 869T2 inoculation in plants (Fig. 3U and V). These data suggest that the 869T2 inoculation in *Arabidopsis* plants might mitigate the salt-induced oxidative stress. Conversely, the mitogen-activated protein kinase (MAPK) signaling pathway gene, *MPK10* gene, showed higher transcript levels in both 869T2-treated and 869T2-treated with salt plants compared to their respective controls (Fig. 3W), indicating the possible regulation of the MAPK signaling pathway by the endophytic bacteria 869T2 inoculation in plants under salt stress.

### After inoculations of the strain 869T2, *Arabidopsis* plants showed better growth under the drought stress

**Fig. 4:**
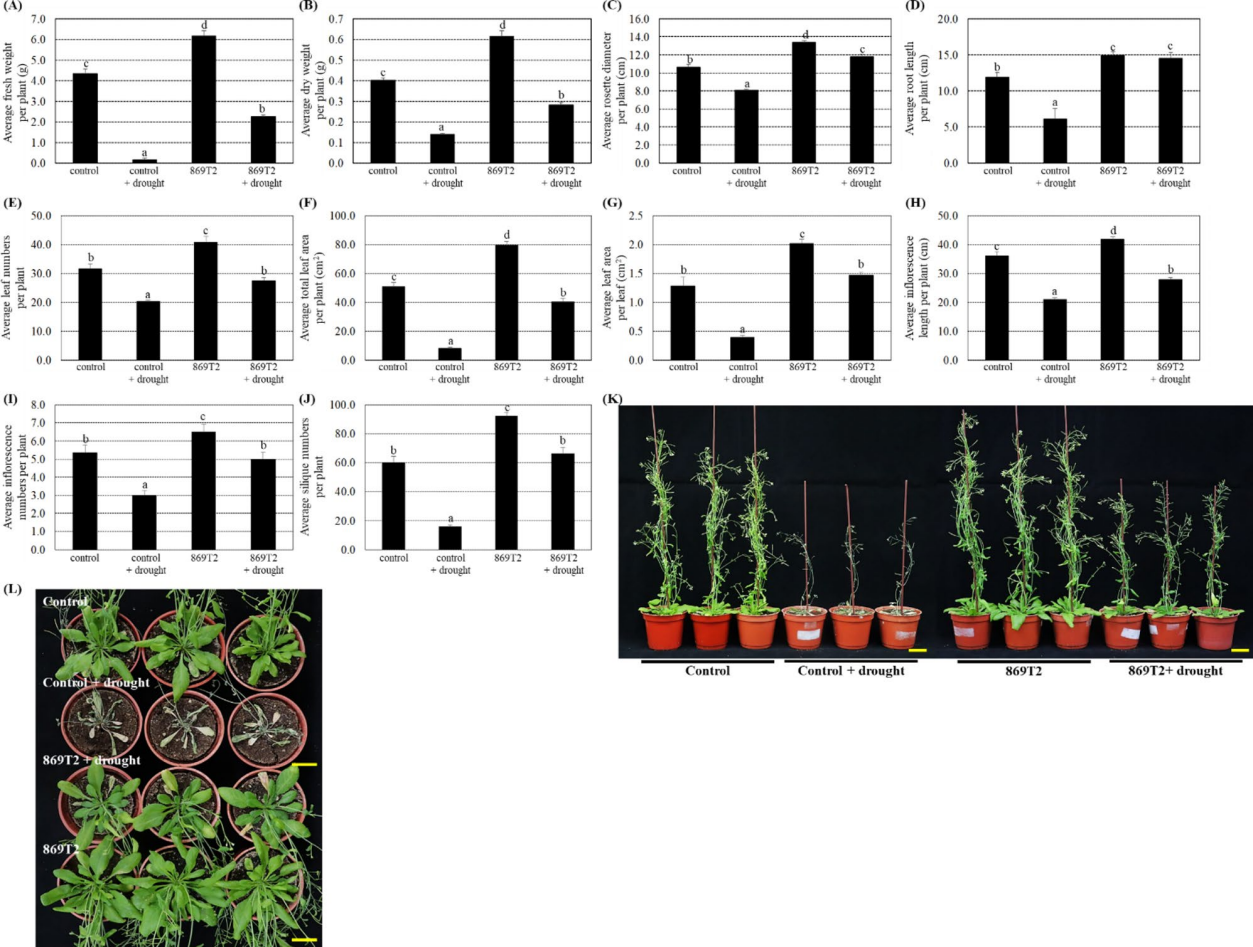
Under drought stress treatments, the 869T2-inoculated *Arabidopsis* plants exhibited better growth than the mock-inoculated control plants. The strain 869T2 was cultured in the LB media and used to inoculate *Arabidopsis* plants. After bacteria inoculations, the plants were treated with drought for ten days by withholding water and subsequently re-watered for three days for recovery. After drought stress treatments, the average fresh weight per plant (Panel **A**), the average dry weight per plant (Panel **B**), the average rosette diameter per plant (Panel **C**), the average root length per plant (Panel **D**), the average leaf numbers per plant (Panel **E**), the average total leaf area per plant (Panel **F**), the average leaf area per leaf (Panel **G**), the average inflorescence length per plant (Panel **H**), the average inflorescence numbers per plant (Panel **J**), and the average silique numbers per plant (Panel **J**) of the control and the 869T2-inoculated plants under drought stress and non-stress conditions were examined. Data are mean±SE (standard error) from at least three independent bacteria inoculation experiments. More than 20 individual plants were examined for each bacteria inoculation assay. Data were analyzed by Duncan tests and means with different letters were significantly different (*p* < 0.05). The side-view (Panel **K**) and top-view (Panel **L**) photographs of the mock-inoculated control and the 869T2-inoculated plants after drought stress treatments. Yellow bar = 3 cm

Given the increased salt stress tolerance in 869T2-inoculated *Arabidopsis*, we examined further if strain 869T2 could help plants grow better under different abiotic stresses. We investigated if inoculation with the strain 869T2 in plants could improve their drought tolerance. The control and 869T2-inoculated *Arabidopsis* plants were subjected to a 10-day drought stress period followed by a 3-day recovery with re-watering. Figure 4 results showed that various plant growth parameters, including the average fresh weight, dry weight, rosette diameter, root length, leaf numbers, total leaf area per plant, leaf area per leaf, inflorescence length and numbers, and silique numbers, were significantly affected by drought stress in both groups. Conversely, under drought stress, the inoculations of the 869T2 in plants had more than 1.4-fold augments in different plant growth parameters when compared with control plants (Fig. 4). These data indicate that the strain 869T2 improved plant growth under drought stress.

We also examined the effects of another abiotic stress treatment on plant growth after 869T2 inoculations. The control and 869T2-inoculated *Arabidopsis* plants were exposed to UV-C lamps (253 nm) for 40 min and subsequently recovered for seven days. Figure S1 showed that values of different plant growth parameters of both control and 869T2-inoculated *Arabidopsis* plants were significantly decreased after exposure to UV-C. However, the 869T2-inoculated plants were heavier and had more healthy leaves than control plants (Figure S1), indicating that the 869T2 inoculations in plants helped plants grow better under UV stress conditions.

### Under salt and drought stresses, the hydrogen peroxide (H_2_O_2_), electrolyte leakage (EL), malondialdehyde (MDA), and proline concentrations were less induced in the 869T2-inoculated *Arabidopsis* plants

**Fig. 5:**
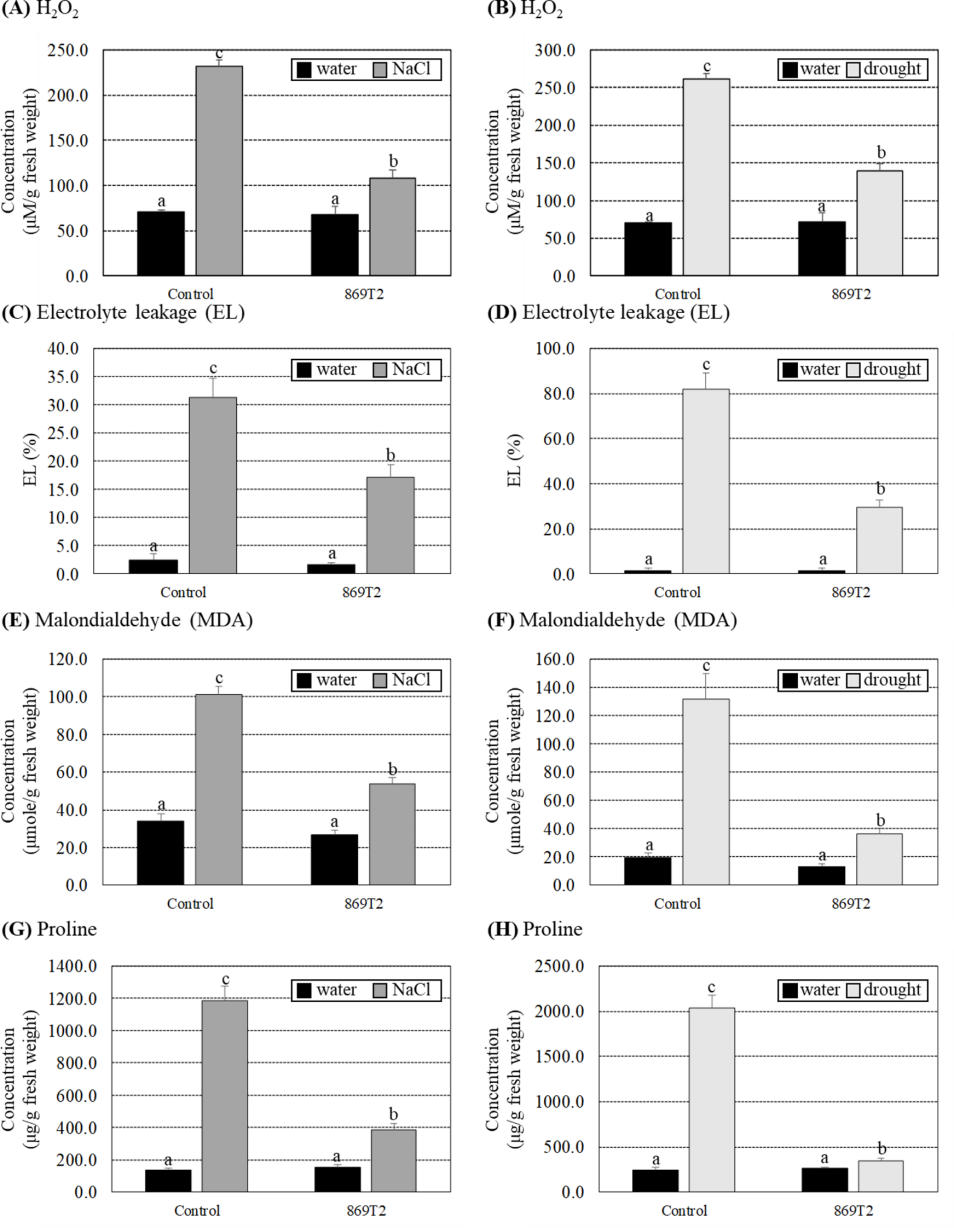
The hydrogen peroxide (H_2_O_2_), electrolyte leakage (EL), malondialdehyde (MDA), and proline concentrations were less induced in the 869T2-inoculated *Arabidopsis* plants under salt and drought stresses. The H_2_O_2_ concentrations (Panel **A**, **B**), electrolyte leakage (EL) (Panel **C**, **D**), MDA contents (Panel **E**, **F**), and proline contents (Panel **G**, **H**) were determined in the mock-inoculated control and the 869T2-inoculated plants under salt (Panel **A**, **C**, **E**, **G**) and drought (Panel **B**, **D**, **F**, **H**) stress treatments. Data are mean±SE (standard error) from at least three independent bacteria inoculation experiments. More than 10 individual plants were examined for each bacteria inoculation assay. Data were analyzed by Duncan tests and means with different letters were significantly different (*p* < 0.05)

Since several stress-responsive genes were up-regulated in 869T2-inoculated *Arabidopsis* plants under salt stress, several biochemical parameters of the control and the 869T2-inoculated *Arabidopsis* plants under salt or drought stresses were determined. Under non-stress conditions, no significant differences in H_2_O_2_, electrolyte leakage (EL), and malondialdehyde (MDA) concentrations were observed between control and 869T2-inoculated plants (Fig. 5A-F). Under salt stresses, the H_2_O_2_ content increased 3.1-fold in the control plants, whereas it only increased 1.6-fold in the 869T2-inoculated plants (Fig. 5A). Under drought stresses, H_2_O_2_ levels increased 3.7-fold in control plants but only 2.0-fold in 869T2-inoculated plants (Fig. 5B). In addition, under both salt and drought treatments, the EL and MDA concentrations were significantly increased in the control plants, but the increased fold was lower in the 869T2-inoculated plants under the same stresses (Fig. 5C-F). When plants grow under salt or drought stress, they can accumulate different metabolites, such as proline and other active molecules, inside plant cells to improve their stress tolerance. Figure 5G and H results showed that proline concentrations in the control plants increased more than 8-fold under salt and drought stresses, but they only increased 1.5- to 2.5-fold in the 869T2-inoculated plants under the same stresses. These data demonstrated that the 869T2-inoculated plants experience less oxidative stress damage than the control plants under salt and drought stresses, consistent with the transcriptome analysis data.

### Improved growth of pak choi (*Brassica rapa*) plants under salt and drought stresses after inoculation with strain 869T2

**Fig. 6:**
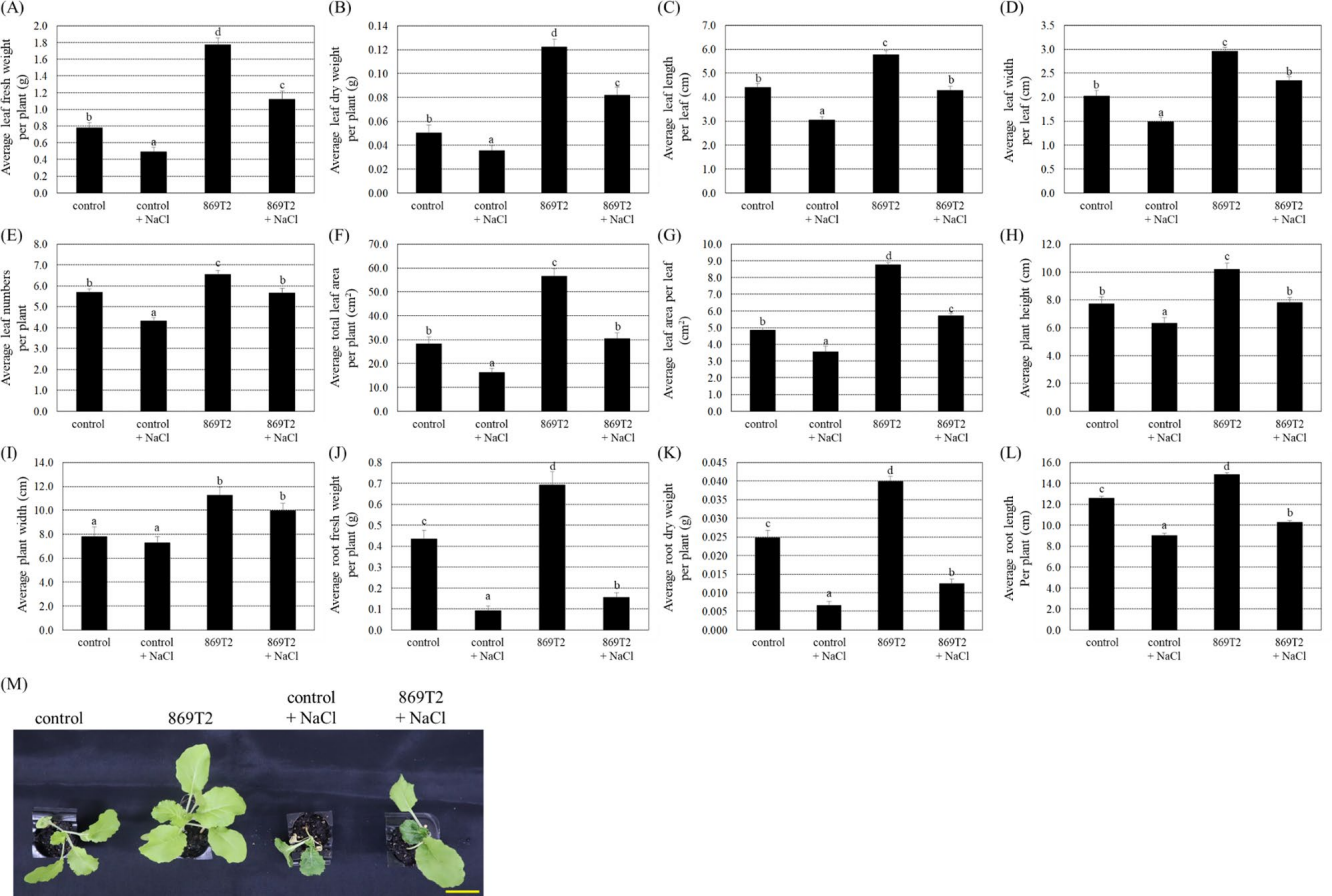
Under the salt stress, strain 869T2 enhanced various growth parameters of the pak choi (*Brassica rapa*) plants compared to the mock-inoculated control plants. After 869T2 inoculations of pak choi plants, plants were treated with the 200 mM NaCl solutions for five days to create the salt stress and then recovered for three days by re-watering with distilled water. After salt stress treatments, the average leaf fresh weight per plant (Panel **A**), the average leaf dry weight per plant (Panel **B**), the average leaf length per leaf (Panel **C**), the average leaf width per leaf (Panel **D**), the average leaf numbers per plant (Panel **E**), the average total leaf area per plant (Panel **F**), the average leaf area per leaf (Panel **G**), the average plant height (Panel **H**), the average plant width (Panel **I**), the average root fresh weight per plant (Panel **J**), the average root dry weight per plant (Panel **K**), the average root length per plant (Panel **L**), and the top-view (Panel **M**) photographs of the mock-inoculated control and the 869T2-inoculated plants under salt stress and non-stress conditions were documented. Data are mean±SE (standard error) from at least three independent bacteria inoculation experiments. More than 20 individual plants were examined for each bacteria inoculation assay. Data were analyzed by Duncan tests and means with different letters were significantly different (*p* < 0.05). Yellow bar = 3 cm

The *B. seminalis* strain 869T2 has been shown to improve the growth of several leafy vegetables, including pak choi (*Brassica rapa* L. R. Chinensis Group) (Hung et al. 2023 and Hwang et al. 2022). We tested if the strain 869T2 could enhance stress tolerance in another eudicot plant from the Brassicaceae family. We treated four-leaf plant seedlings of pak choi with 200 mM NaCl for five days to create salt stress and allowed the seedlings to recover for three days by watering with distilled water. After salt stress treatment, fresh and dry weights of both leaves and roots and other growth parameters were lower in control and 869T2-inoculated plants compared to their respective non-stressed counterparts (Fig. 6). These data suggest that salt stress treatments greatly hindered pak choi plant growth. Under salt stress treatments, the average values of the leaf fresh and dry weight, leaf length and width, leaf numbers per plant, total leaf area per plant, leaf area per leaf, plant height, plant width, root fresh and dry weight, and root length were significantly more prominent in the 869T2-inoculated pak choi in comparison to that of the control plants (Fig. 6). The data demonstrated that the inoculation of the endophytic bacteria strain 869T2 in pak choi plants enhanced its salt tolerance.

**Fig. 7:**
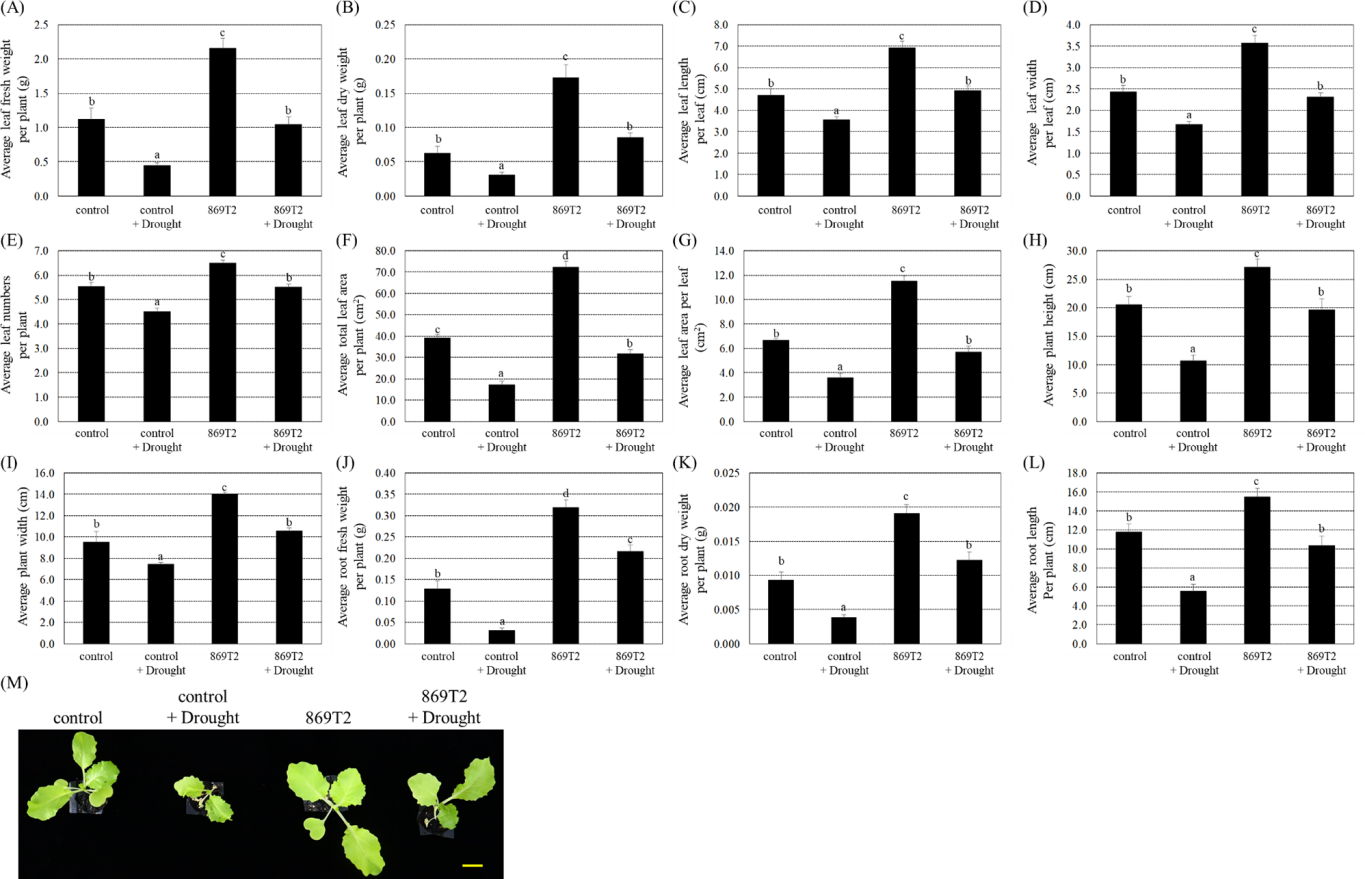
Under drought stress, the pak choi (*Brassica rapa*) plants became larger and heavier after inoculations of the strain 869T2. After bacteria inoculation of pak choi plants, plants were treated with drought for five days by withholding water and subsequently re-watered for three days for recovery. After drought stress treatments, the average leaf fresh weight per plant (Panel **A**), the average leaf dry weight per plant (Panel **B**), the average leaf length per leaf (Panel **C**), the average leaf width per leaf (Panel **D**), the average leaf numbers per plant (Panel **E**), the average total leaf area per plant (Panel **F**), the average leaf area per leaf (Panel **G**), the average plant height (Panel **H**), the average plant width (Panel **I**), the average root fresh weight per plant (Panel **J**), the average root dry weight per plant (Panel **K**), the average root length per plant (Panel **L**), and the top-view (Panel **M**) photographs of the mock-inoculated control and the 869T2-inoculated plants under drought stress and non-stress conditions were determined. Data are mean±SE (standard error) from at least three independent bacteria inoculation experiments. More than 20 individual plants were examined for each bacteria inoculation assay. Data were analyzed by Duncan tests and means with different letters were significantly different (*p* < 0.05). Yellow bar = 3 cm

Additionally, the pak choi seedlings were also subjected to drought stress by withholding water for five days, followed by a 3-day recovery period. Under drought stresses, average values of several plant growth parameters in the control plants and the 869T2-inoculated plants were lower than those of the respective plants without drought stress treatments (Fig. 7), indicating that dehydration treatments substantially decreased plant growth. Under drought stresses, the 869T2-inoculated pak choi plants had relatively higher values of various plant growth parameters than the control plants (Fig. 7). These data suggest that inoculation with the bacteria strain 869T2 improved plant tolerance to drought stress.

**Fig. 8:**
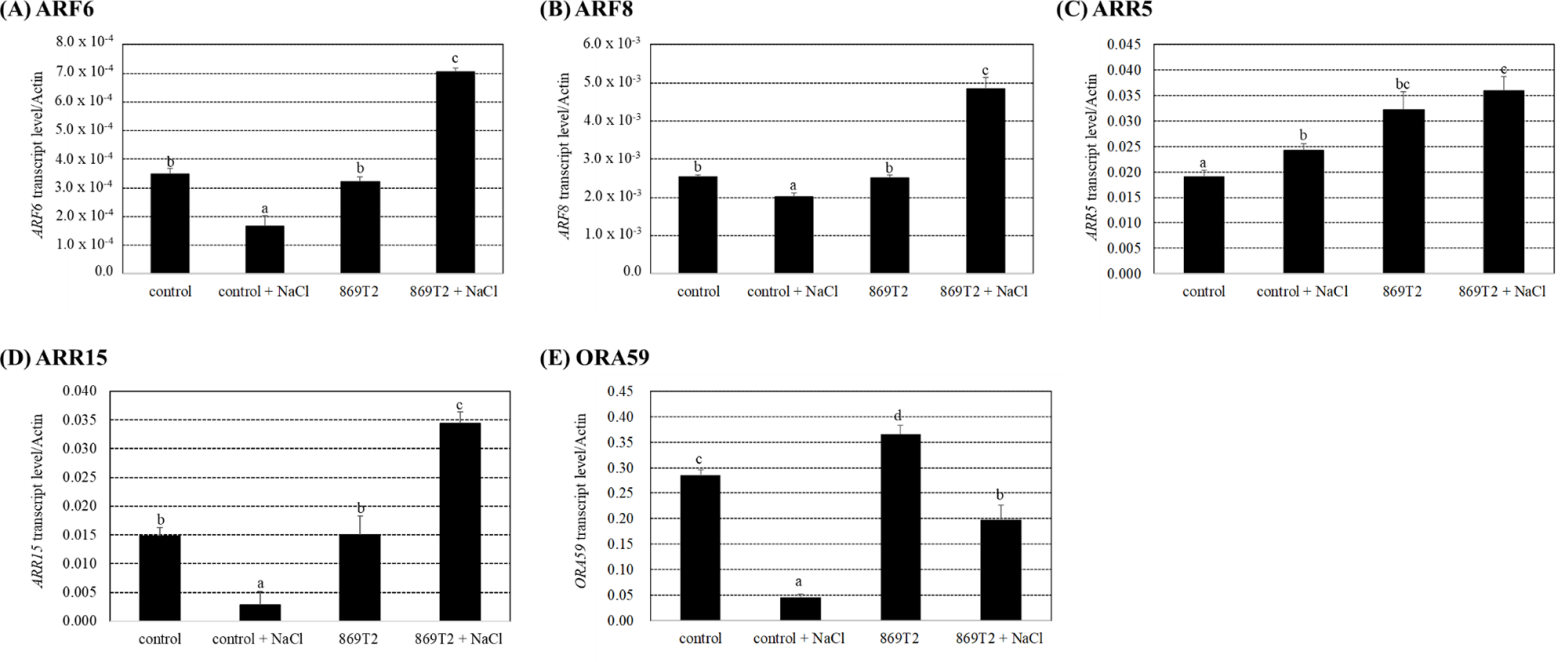
The induction levels of hormone response-related gene expression were different in the mock-inoculated control and the 869T2-inoculated pak choi (*Brassica rapa*) plants under non-stress and salt stress conditions. Transcript levels of the auxin-responsive genes: *ARF6* (Panel **A**), *ARF8* (Panel **B**), the cytokinin response regulators: *ARR5* (Panel **C**), *ARR15* (Panel **D**), and the ethylene-responsive gene: *ORA59* (Panel **E**) in control and 869T2-inoculated pak choi plants with and without salt treatments were measured by qPCR analysis. The *Actin-2* transcript level was an internal control. Data are mean±SE from at least three independent biological experiments. Data were analyzed by Duncan test, and means with different letters were significantly different (*p* < 0.05)

Both the transcriptomic and qPCR results demonstrated that several genes related to auxin, cytokinin, and ethylene signaling pathways were differentially expressed in *Arabidopsis* plants after 869T2 inoculation under salt stress (Figs. 2F and 3). We therefore examined several phytohormone signaling pathway-related gene expression levels by qPCR in pak choi plants after the strain 869T2 inoculations with and without salt treatments. The qPCR results showed that the auxin signaling-related genes, *AUXIN RESPONSE FACTOR 6 (ARF6)* and *ARF8*, the cytokinin response regulators, *ARR5*, and *ARR15*, and the ethylene signaling response-related gene, *OCTADECANOIC-RESPONSIVE ARABIDOPSIS 59/ERF59 (ORA59)*, were significantly induced after the 869T2 inoculation and salt treatments in comparison to the control with salt treatments (Fig. 8). These results are consistent with those obtained in *Arabidopsis* plants, indicating enhanced phytohormone signaling responses in 869T2-inoculated pak choi under salt stress.

**Fig. 9:**
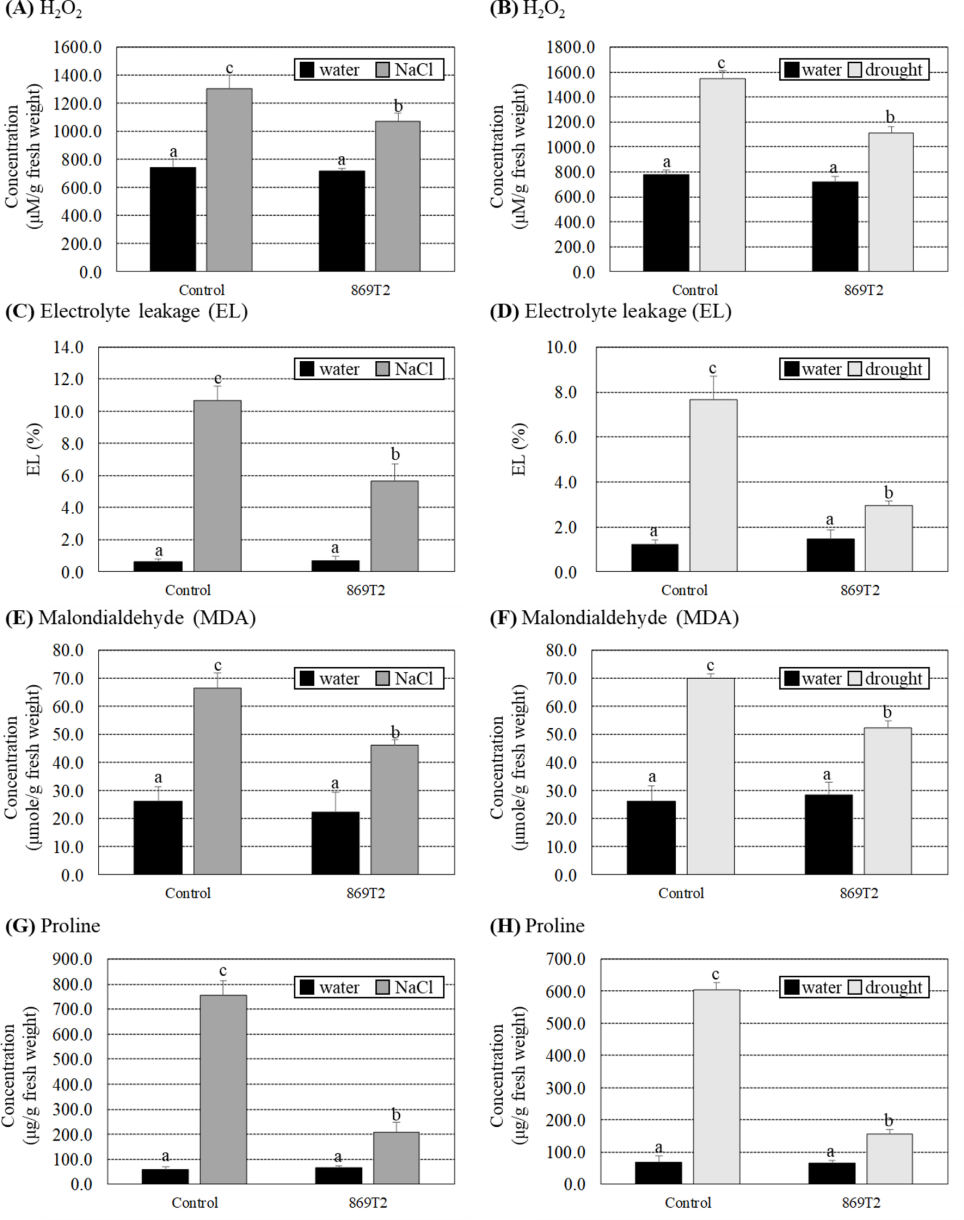
The 869T2-inoculated pak choi (*Brassica rapa*) plants had lower induced H_2_O_2_, electrolyte leakage, malondialdehyde, and proline concentrations than the mock-inoculated control plants under salt and drought stresses. The H_2_O_2_ concentrations (Panel **A**, **B**), electrolyte leakage (EL) (Panel **C**, **D**), MDA contents (Panel **E**, **F**), and proline contents (Panel **G**, **H**) were recorded in the mock-inoculated control and the 869T2-inoculated plants under salt (Panel **A**, **C**, **E**, **G**) and drought (Panel **B**, **D**, **F**, **H**) stress treatments. Data are mean±SE (standard error) from at least three independent bacteria inoculation experiments. More than 10 individual plants were examined for each bacteria inoculation assay. Data were analyzed by Duncan tests and means with different letters were significantly different (*p* < 0.05)

After salt and drought stress treatments, several biochemical parameters of H_2_O_2_, EL, MDA, and proline concentrations in the control and 869T2-inoculated pak choi seedlings were then determined. The H_2_O_2_, EL, MDA, and proline contents were not significantly different between the control and the 869T2-inoculated pak choi plants under non-stress conditions (with water treatments) (Fig. 9). Under NaCl treatments, the H_2_O_2_, EL, MDA, and proline contents increased 1.7- to 13.0-fold in the control plants (Fig. 9A, C, E and G). On the contrary, in the 869T2-inoculated pak choi plants, the H_2_O_2_, EL, MDA, and proline contents only increased 1.4- to 3.3-fold under salt stress treatments (Fig. 9A, C, E and G). When pak choi plants were treated with drought stress, the increase in these parameters was lower in 869T2-inoculated plants compared to control plants (Fig. 9B, D, F and H). In summary, these results indicated that inoculation of the pak choi seedlings with strain 869T2 enhanced plant growth and simultaneously reduced various oxidative stress damages caused by salt and drought stresses.

### Enhanced growth of Chinese amaranth (*Amaranthus tricolor*) plants under salt and drought stresses following inoculation with strain 869T2

We also tested the Chinese amaranth (*Amaranthus tricolor*) of the Amaranthaceae family to examine the effect of strain 869T2 on its growth under salt and drought stresses. Four-leaf seedlings of Chinese amaranth (*Amaranthus tricolor*) were subjected to salt stress by treating them with 250 mM NaCl for seven days, followed by a recovery period of three days using distilled water irrigation. Following salt stress, the fresh and dry weights of leaves and roots, along with other growth parameters, were reduced in both the control and *Burkholderia seminalis* strain 869T2-inoculated plants compared to their respective non-stressed counterparts (Figure S2). These findings indicate that salt stress significantly inhibited the growth of Chinese amaranth plants.

Notably, under salt stress conditions, 869T2-inoculated plants exhibited significantly higher values for key growth parameters, including leaf fresh and dry weights, leaf length and width, number of leaves per plant, total leaf area per plant, leaf area per leaf, plant height, plant width, root fresh and dry weights, and root length, compared to non-inoculated control plants (Figure S2). These results demonstrate that inoculation with the endophytic bacterial strain 869T2 substantially enhanced the salt tolerance of Chinese amaranth seedlings. Additionally, the data further underscore the potential of 869T2 to confer improved resilience against salt stress in plants, as previously observed in pak choi and *Arabidopsis*.

Chinese amaranth seedlings were subjected to drought stress by withholding water for seven days, followed by a three-day recovery period under normal watering conditions. Drought stress resulted in a significant reduction in key growth parameters, including fresh and dry weights, leaf dimensions, and root metrics, in both the control and *Burkholderia seminalis* strain 869T2-inoculated plants compared to their respective non-stressed counterparts (Figure S3). These findings indicate that dehydration treatments caused substantial growth inhibition in Chinese amaranth seedlings. However, under drought stress conditions, 869T2-inoculated plants consistently exhibited higher values across multiple growth parameters, including fresh and dry biomass of leaves and roots, leaf number, leaf area, and root length, in comparison to non-inoculated control plants (Figure S3). These results demonstrate that inoculation with *B. seminalis* strain 869T2 enhanced the drought tolerance of Chinese amaranth seedlings, mitigating the adverse effects of water deficit on plant growth. This highlights the potential of strain 869T2 as a beneficial endophyte for improving plant resilience to drought stress.

## Discussion

Over the past years, climate change has become one of the most severe environmental distresses. As a result of climate change and global warming, crop plants are exposed to a wide range of environmental stresses, which in turn hinder crop yield and productivity. As the temperatures rise, it causes more water evaporation, reduces water availability, increases salt deposits in soils, and dries off soils and vegetation. Both salt and drought are the most common and complex abiotic stress factors for plants. There is an increasing need to develop sustainable and low-environmental-impact alternative strategies to cope with the negative impacts of salt and drought stresses on plant growth. Using beneficial endophytic bacteria can significantly improve abiotic stress tolerance and offer additional benefits, including enhancing plant growth, increasing nutrient acquisition, diminishing plant disease occurrences, and consequently decreasing usage of chemical fertilizers and pesticides. This study successfully demonstrated that the endophytic bacteria strain 869T2 assisted *Arabidopsis*, pak choi, and Chinese amaranth plants to grow better and larger after salt and dehydration treatments. Our transcriptomic and qPCR results showed that inoculation with the 869T2 significantly enhanced the salt stress tolerance of the *Arabidopsis* plant by modulating various plant hormone response pathways and stress-responsive gene expressions. Furthermore, these 869T2-inoculated plants had relatively lower induced H_2_O_2_, EL, MDA, and proline contents than the control plants under salt and drought stress treatments, which correlated well with the better growth and development shown in these 869T2-inoculated plants. This multifaceted response suggests a complex interaction between the bacterium 869T2 and plant hormonal signaling responses that ultimately improve plant resistance under abiotic stress conditions.

Various phytohormones are involved in the decrease of abiotic stresses in plants. The indole-3-acetic acid (IAA) is one of the crucial phytohormones produced in plants, regulates cell divisions, cell elongations and differentiations, root, leaf, and fruit development, photosynthesis, vascular development, phototropism, senescence, and has great control of different plant growth and development process (Zhang et al. 2022). The plant endophytic bacteria can contribute to biomass accumulation and assist more nutrient acquisition of host plants by producing or regulating various phytohormones under stress conditions (Verma et al. 2021; Watts et al. 2023). In this study, the endophytic bacteria *Burkholderia seminalis* strain 869T2 can produce IAA in the presence of tryptophan (Hwang et al. 2021) and improv plant growth under salt stress by up-regulating various auxin-response gene expressions. The endophytic bacteria-produced IAA may be assimilated by plant cells that could affect the auxin signal transduction pathway in plants and stimulate plant cell proliferation and growth under stress conditions (Krishnamoorthy et al. 2022; Lachu et al. 2022; Malik and Arora 2022). Our qPCR analysis demonstrated a significant up-regulation of auxin-responsive genes (*GH3.1*, *IAA15*, *ARF*, and *SAUR*) in plants inoculated with the 869T2 compared to the control. In plants, the auxin signaling pathways are controlled by three core proteins: TRANSPORT INHIBITOR RESPONSE 1/AUXIN SIGNALING F-BOX PROTEIN (TIR1/AFB) auxin coreceptors, Auxin/INDOLE-3-ACETIC ACID (Aux/IAA) transcriptional repressors, and the AUXIN RESPONSE FACTOR (ARF) transcription factors. The *IAA*, *SAUR*, and *GRETCHEN HAGEN3* (*GH3*) genes are rapidly induced in response to auxin (Zhang et al. 2022). Many *SAUR* gene expression levels are regulated by multiple hormonal and environmental signals, including abiotic stress conditions, and are involved in promoting cell expansion (Guo et al. 2018; Zhang et al. 2021). The observed increase in *SAUR47*, *SAUR52*, *SAUR57*, and *SAUR69* transcript levels in the 869T2 inoculated plants with salt treatments further suggests the role of these genes in enhancing stress tolerance through auxin signaling modulation. The auxin signaling repressor IAA15 was identified as a substrate of the mitogen-activated protein kinase, which can suppress later root development and extend the long primary roots into the deeper layers of soils to reach more available underground water under drought stress (Kim et al. 2022). Furthermore, the *GH3* gene family encodes a class of auxin-induced conjugating enzymes that regulate the levels of active auxin. Specifically, the *GH3.1* and other group II and III *GH3* genes are known to be up-regulated under salt stress, modulating auxin levels to mitigate stress effects (Bahieldin et al. 2018).

Like our observations, the *Burkholderia phytofirmans* strain PsJN, isolated from onion roots, showed higher IAA productions and increased host plant abiotic stress tolerances, including chilling, drought, and salt stresses (Fernandez et al. 2012; Naveed et al. 2014, 2015; Yang et al. 2020). Furthermore, the *Arabidopsis* transcriptome analysis results revealed that the auxin-related genes, *IAA1* and *SAUR68*, were induced after the *B. phytofirmans* strain PsJN inoculations in *Arabidopsis* (Poupin et al. 2013). Another rice endophytic bacteria, *Burkholderia kururiensis*, produced IAA, enhanced rice plant growth and grain yield, and induced IAA-responsive gene expressions in transgenic rice roots (Mattos et al. 2008). An endophytic bacteria *Staphylococcus* sp., isolated from the rhizome of *Curcuma longa*, produced IAA and provided plant-promotion effects of *Vigna unguiculata* under drought conditions (Jayakumar et al. 2020). A previous study showed that IAA-producing bacteria colonizing the root nodules of wild legumes enhanced drought tolerance after inoculations in plants (Cardoso et al. 2018). Inoculating wheat seedlings with the IAA-producing *Azospirillum lipoferum* strains increased leaf water content during drought stress and enriched root development to obtain more water and nutrients (Arzanesh et al. 2011). Another IAA-producing *Bacillus velezensis* FMH2 enhanced plant root and lateral root development, improving tomato salinity tolerance (Masmoudi et al. 2021). Previous studies, along with our observations, suggest that the *B. seminalis* 869T2 strain may be similar to other *Burkholderia* species and other plant growth-promoting bacteria to utilize bacterially produced IAA and regulate the auxin signaling pathways in host plants under adverse conditions to sustain host plant growth and mitigate stress impacts.

The up-regulation of cytokinin response regulators (*ARR5*, *ARR15*, and *ARR19*) and the gibberellin biosynthesis gene *GA20OX2* in the 869T2 and salt stress-treated plants underscores the roles of the strain 869T2 in enhancing cytokinin and gibberellin pathways of host plants under the abiotic stress. Cytokinin (CK) is a significant growth-promoting phytohormone that plays diverse roles in different aspects of plant cell growth and development, including cell division and enlargement, tissue expansion, nutrient allocation, vascular development, and biotic and abiotic responses (Chieb and Gachomo 2023; Watts et al. 2023). Some plant growth-promoting bacteria have been reported to produce cytokinin and enhance the abiotic stress tolerance of host plants (Chieb and Gachomo 2023; Watts et al. 2023). The cytokinin-producing *Bacillus subtilis* increased the lettuce cytokinin content and improved plant growth under drought stress (Arkhipova et al. 2005). Another CK-producing bacterium, *Azospirillum brasilense* RA − 17, increased wheat growth and yield, the photosynthetic rates and chlorophyll contents, and antioxidant enzyme activities in inoculated plants (Zaheer et al. 2022). The CK-producing *Bacillus subtilis* also improved the drought tolerance of *Platycladus orientalis* (oriental thuja) (Liu et al. 2013). The *Bacillus subtilis*-inoculated plants improved relative water content, increased root and shoot dry weights, and enhanced production of root exudates under drought conditions (Liu et al. 2013). The plant growth-promoting bacteria, *Bacillus velezensis* 83 (*Bv* 83), enhanced *Arabidopsis* root and shoot biomass production, induced expression of cytokinin-related gene *ARR5*, and its growth promotion ability required the proper functions of cytokinin receptors CRE1/AHK4, AHK2 and AHK3 (Barrera-Ortiz et al. 2023).

Gibberellin (GA) is another major phytohormone that regulates plant growth and development, from seed germination, vegetative growth, stem elongation, and flowering, to fruit and seed set (Verma et al. 2021; Watts et al. 2023). One of the GA biosynthetic genes, *GA3OX1* (Gibberellin 3-beta-dioxygenase), was induced in the *B. phytofirmans* strain PsJN-inoculated *Arabidopsis* plants and may promote vegetative growth in host plants (Poupin et al. 2013). A GA-producing endophytic bacterial strain, *Bacillus amyloliquefaciens*, from rice seeds, colonized the rice roots and improved rice plant growth (Shahzad et al. 2016). Results from our study and previous studies have further supported the idea that phytohormones CK and GA play important roles in the plant growth promotion abilities of plant endophytic bacteria. Our data suggest that inductions of CK and GA signaling pathways in the 869T2-inoculated plants could support growth under optimal conditions and may help plants cope with stress by regulating different hormone responses.

Ethylene, a simple gaseous phytohormone, enhances plant tolerance to salt stress by maintaining the homeostasis of Na^+^/K^+^, nutrients, and reactive oxygen species (ROS) through stimulating antioxidant defense responses (Gao et al. 2022; Riyazuddin et al. 2022; Verma et al. 2021). Furthermore, overproduction of endogenous ethylene or treatments of exogenous ethylene-releasing compounds improves salt stress tolerance in different plants (Riyazuddin et al. 2022; Shekhawat et al. 2022). The ethylene signaling response genes (*ERS2*, *ERF1*, *ERF14*, *ERF110*, and *ORA59*) were up-regulated in 869T2-treated plants, with *ERF1*, *ERF14*, and *ORA59* showing higher expression under the salt stress compared to the respective control. These data were consistent with previous studies showing that the ethylene signaling pathway in plants may mediate stress responses and improve plant resilience under stress conditions (Riyazuddin et al. 2022; Shekhawat et al. 2022). Abscisic acid (ABA), a stress-responsive hormone, maintains the water potential inside plant cells by lowering transpiration activity via stomatal closure and activates various stress resistance genes under water deficit or salt stress (Chieb and Gachomo 2023; Ilangumaran and Smith 2017; Watts et al. 2023). Under abiotic stress conditions, the ABA level increases and is perceived by a group of receptors that are members of Pyrabactin Resistance1/PYR1-like/Regulatory Components of ABA Receptors (PYR1/PYL/RCARs, referred as PYLs) family (Chieb and Gachomo 2023; Watts et al. 2023). In our study, the higher expressions of the ABA signaling response-related genes, *PYL11*, *ABI5*, and *CBF2*, in the strain 869T2 and salt stress-treated plants, suggesting that the inoculations of the 869T2 may enhance ABA pathways and may contribute to the improved stress tolerance. Another study has shown increased transcript levels of *CsPYL1* and *CsPYL2* in leaves of *Bacillus methylotrophicus*-treated cucumber plants under drought stress (Hou et al. 2018). The CBF transcription factors interact with promoters of stress-responsive genes, contributing to plant tolerance to cold and salt stress by modulating different gene expressions (Yan et al. 2023; Zhao et al. 2016; Zhao and Zhu 2016). A seed-borne bacterial endophyte, *Bacillus amyloliquefaciens* RWL-1, produced ABA under saline conditions and could potentially increase salt tolerance in inoculated rice plants (Shahzad et al. 2017). Other plant growth-promoting bacteria can induce plant tolerance to abiotic stresses by affecting ABA contents and/or ABA-related responses (Chieb and Gachomo 2023; Kour and Yadav 2022). The beneficial strain, *Phyllobacterium brassicacearum* STM196, increased ABA amounts in the STM196-inoculated *Arabidopsis* plants, which subsequently decreased leaf transpiration and improved osmotic stress tolerance in host plants (Bresson et al. 2013). The endophytic bacteria *Azospirillum lipoferum* USA59b produced ABA that may alleviate the adverse effects of drought stress in maize plants (Cohen et al. 2009). The *Bacillus marisflavi* CRDT-EB-1 produced the ABA precursors, xanthoxin and xanthoxic acid, induced stomatal closure, and improved drought stress tolerance of inoculated mustard seedlings (Gowtham et al. 2021). Our study and other similar studies have demonstrated the importance of ethylene and ABA in ameliorating salt and drought tolerance of host plants by the endophytic bacteria.

The salt and drought stresses induce reactive oxygen species (ROS) production, including hydrogen peroxide (H_2_O_2_), superoxide radicals, and hydroxyl radicals, which disturb cellular redox homeostasis, cause oxidative stress, damage nucleic acids, degrade membrane proteins, peroxidize lipids, deteriorates membrane integrity, enhances electrolyte leakage, hampers enzyme activity, causes cell damage and potentially cell death (Gao et al. 2022; Kour and Yadav 2022; Krishnamoorthy et al. 2022; Vaishnav et al. 2019). Under stress, malondialdehyde (MDA) is generated by the lipid peroxidation of the plasma membrane by ROS. The *PRX33* and *PRX34* genes encode extracellular peroxidases that contribute to H_2_O_2_ generations and play significant roles in the oxidative burst in response to biotic and salt stresses (Kámán-Tóth et al. 2019; Liu et al. 2021; Szymańska et al. 2019). The sharp decrease in transcript levels of ROS-related genes (*PRX33* and *PRX34*) in the 869T2 and salt stress-treated plants suggests that the 869T2 inoculation may mitigate oxidative stress. The reduced expression of these genes indicates a lower accumulation of reactive oxygen species, which is beneficial for maintaining cellular integrity under stress. Consistent with the lower *PRX* gene expressions, the 869T2-inoculated plants had relatively lower induced H_2_O_2_, EL, and MDA concentrations than the control plants under salt and drought stresses. In accordance with our observations, several endophytic bacteria, including *Priestia megaterium* BP-R2, *Bacillus megaterium* HX-2, *Bacillus amyloliquefaciens* SB-9, and *Burkholderia phytofirmans* PsJN, have been demonstrated to increase plant tolerance to salt or drought stress by inducing stress-associated enzyme expression, reducing ROS accumulation, and/or decreasing electrolyte leakage (EL) and MDA contents in host plants (Hwang et al. 2022; Jiao et al. 2016; Li et al. 2019; Sheibani-Tezerji et al. 2015). In conclusion, previous studies and our findings suggested that abilities of IAA productions, regulations of various host hormone-response and stress-responsive gene expressions, and reduction of oxidative stress in the strain 869T2 may collectively contribute to the salt and drought stress tolerances of *Arabidopsis* and pak choi plants. Furthermore, our study has revealed the possible application of strain 869T2 as a bioinoculant to improve abiotic stress tolerance in sustainable agricultural practices.

## Materials and methods

### Inoculation of plants with bacteria, abiotic stress treatments, and measurement of plant growth parameters

The bacteria inoculation of plants and re-isolation assays were conducted according to the protocols of Hwang et al. (2021, 2022). The four- to six-leaf seedlings of wild-type *Arabidopsis thaliana* (ecotype Columbia), pak choi (*Brassica rapa* L. R. Chinensis Group), and Chinese amaranth (Amaranthus tricolor) grown in soils (BVB substrates, Maasland, Nederland) were inoculated with the bacteria *B. seminalis* strain 869T2 (Hwang et al. 2021). The 869T2 cells were grown on LB media (pH 7.5) at 30 °C to an approximate OD_600_ value of 0.6–0.8. Bacterial cultures were then collected and adjusted to approximately 10^8^ cfu mL^− 1^, as determined by plate counting. Five milliliters of bacterial culture were added to the potting soils of each plant. After bacteria inoculation, plant seedlings were continually grown in pots for 10–14 days for *Arabidopsis* and for 3–4 days for pak choi and Chinese amaranth plants, followed by abiotic stress treatments. Plant tissues were surface-sterilized and ground with sterile distilled water to verify the endophytic colonization of the inoculated plants with the strain 869T2. The plant crude extracts were then serially diluted and plated on LB agar to determine the viable bacteria cell numbers. Bacteria were identified via sequencing and phylogenetic analysis of the 16 S ribosomal RNA (16 S rRNA) gene. The 16 S rRNA gene was amplified by colony PCR reactions with primers E8F (5′-AGAGTTTGATCATGGCTCAG-3′) and U1510R (5′-CGGTTACCTTGTTACGACTT-3′) (Hwang et al. 2021).

The abiotic stress treatments were performed according to Hwang et al. (2022). For the salt stress treatments, *Arabidopsis*, pak choi, and Chinese amaranth plants inoculated with the *B. seminalis* strain 869T2 were subjected to the following conditions: *Arabidopsis* was treated with 250 mM NaCl for five days, pak choi with 200 mM NaCl for five days, and Chinese amaranth with 250 mM NaCl for seven days. Then, the NaCl-treated plants were treated with distilled water to recover for three days. The distilled water was used to treat the negative control plants. For drought stress treatments, the 869T2-inoculated *Arabidopsis*, pak choi, and Chinese amaranth plants were subjected to controlled dehydration by withholding water for ten, five, and seven days, respectively. The plants were then re-watered for three days to recover. For UV-C stress treatments, the 869T2-inoculated *Arabidopsis* plants were exposed to Sankyo Denki 20-w UV-C lamps (253 nm) for 40 min and subsequently recovered for seven days. After various abiotic stress treatments, several plant growth parameters were determined in harvested *Arabidopsis*, pak choi, and Chinese amaranth plants as described previously (Hwang et al. 2021, 2022). The *Arabidopsis*, pak choi, or Chinese amaranth plants that were not inoculated with strain 869T2 and not treated with abiotic stress served as the negative controls.

### *Arabidopsis* plant RNA extraction, transcriptomic analysis, and quantitative real-time PCR (qPCR) analysis

Total RNA was extracted from the control and the 869T2-inoculated *Arabidopsis* plants treated with 250 mM NaCl for five days and subsequently recovered for three days with distilled water. Similarly, total RNA was isolated from the mock-inoculated control and the 869T2-inoculated pak choi plants treated with 200 mM NaCl for five days and then recovered for three days with distilled water. Plant tissues were then ground with liquid nitrogen and mixed with TRIZOL LS reagents (Total RNA Isolation Reagent for Liquid Samples from Invitrogen, Carlsbad, CA, USA) to isolate RNA according to the manufacturer’s instructions. The library was constructed by the Agilent’s SureSelect XT HS2 mRNA Library Preparation Kit, followed by AMPure XP beads (Beckman Coulter, USA) size selection, and performed 75 bp single-end sequencing on Illumina Solexa platform. The sequence was determined with Illumina’s sequencing-by-synthesis (SBS) technology (Illumina, USA). The Illumina’s program bcl2fastq v2.20 was used for basecalling and trimmed off low-quality reads based on Q20 accuracy. All bioinformatics tools were used with default settings according to Welgene Biotech’s (Taipei, Taiwan) in-house pipeline. The resulting sequences were mapped to the TAIR10 genome.

The differential expression analysis was performed using the DESeq2 package on read count data (Love et al. 2014). Low count genes (sum of read counts < 10) were filtered out, and the DESeq normalization method was based on the median of ratios of gene counts was applied to the dataset. Genes with an adjusted p-value less than 0.05 and absolute log2 fold change over one were considered differentially expressed. Enrich KEGG function from the cluster Profiler package (Wu et al. 2021; Yu et al. 2012) was utilized for Kyoto Encyclopedia of Genes and Genomes (KEGG) pathway enrichment analysis. The heatmap for the DEGs was created with R version 4.2.3 (R Statistical Software) using the pheatmap package (https://cran.r-project.org/web/packages/pheatmap/index.html).

The cDNA was obtained by reverse transcribing 1-µg RNA samples using oligo-dT primers. The 100-ng cDNAs were used for quantitative real-time PCR (qPCR) with the IQ^2^ SYBR Green Fast qPCR System Master Mix (Bio-genesis Technologies Inc., Taipei, Taiwan) in an MS3000P QPCR system (Agilent Technologies, Santa Clara, CA, USA). Primers used for qPCR are listed in the Supplementary Table S1. The *UBQ10* (polyubiquitin 10) and *Actin-2* transcript levels were used as internal controls for each qPCR reaction with *Arabidopsis* and pak choi plant samples, respectively. The PCR amplification cycle was 99℃ for 1 min (1 cycle); 94℃ for 30 s, 56℃ for 40 s, 72℃ for 1 min (50 cycles); 99℃ for 1 min (1 cycle); 55℃ for 3 min (1 cycle); and 95℃ for 30 Sect. (1 cycle). More than 3 independent real-time PCR reactions were performed with RNA samples isolated from at least 6–8 different *Arabidopsis* or pak choi plants.

### Hydrogen peroxide (H_2_O_2_), electrolyte leakage (EL), malondialdehyde (MDA), and proline content determination

Leaves from the mock-inoculated control and the 869T2-inoculated, stress-treated plants were collected and used to determine the hydrogen peroxide (H_2_O_2_), electrolyte leakage (EL), malondialdehyde (MDA), and proline contents according to protocols of Huang and Hwang (2020) and Hwang et al. (2022). In brief, the hydrogen peroxide (H_2_O_2_) concentrations were measured by a colorimetric reaction with xylenol orange (XO) and were determined by an absorbance at 560 nm by comparing with a standard curve of 0–100 µM H_2_O_2_.

To determine the electrolyte leakage (EL) amounts of plant samples, the fresh leaves were placed in tubes with deionized water, and the electrical conductance (EC1) was measured after leaf samples were shaken for 24 h at room temperature. Then leaf samples were then subjected to 121 °C for 20 min in an autoclaved oven, and the final electrical conductance (EC2) was measured. The electrical conductance (ECnc) of the deionized water without leaf samples was used as a negative control. The electrolyte leakage (EL) was calculated as follows: EL (%) = (EC1-EC1nc/EC2-EC2nc) × 100.

To determine the MDA content in plants, leaves were homogenized and extracted with 20% (w/v) trichloroacetic acid (TCA) containing 0.5% (w/v) thiobarbituric acid (TBA). The plant mixture was heated at 95 °C for 30 min and quickly cooled in an ice bath. After centrifugation, the absorbances of the plant crude extract were measured at 440 nm, 532 nm, and 600 nm. The MDA concentration was quantified as µmol/g fresh weight. To measure the proline content of plants, leaves were homogenized with 3% sulfosalicylic acid, mixed with the acidic ninhydrin reagent, and heated at 100 °C for one hour. The plant mixture was then extracted with toluene, and the absorbance was read at 520 nm.*w*/*v*

### Statistical analysis

The plant growth measurements were average values from at least three independent bacteria inoculation experiments. At least 15 different *Arabidopsis*, pak choi, or Chinese amaranth plant seedlings were inoculated with the strain 869T2, and more than 60 individual plants were examined for bacteria inoculation assays. Error bars were calculated using the Microsoft Excel (Microsoft Corporation, Redmond, WA, USA) STDEVP function. All statistical analyses were conducted using the IBM SPSS statistical analysis software. Comparisons of different treatments were evaluated using the Duncan test. The statistical significance was noted at a probability level (*p*) < 0.05.

## Electronic supplementary material

Below is the link to the electronic supplementary material.

Supplementary Material 1

## Data Availability

The RNAseq data presented in this article have been submitted to the NCBI Sequence Read Archive and can be accessed using BioProject accession no. PRJNA1127305.
