# Supplementary material for: A plant endophytic bacterium Burkholderia seminalis strain 869T2 increases plant growth under salt stress by affecting several phytohormone response pathways
Source: Bot Stud. 2025 Feb 4;66:7. doi: 10.1186/s40529-025-00453-3 (PMC11794907; doi:10.1186/s40529-025-00453-3)
Supplement: Supplementary file 1 — Supplementary Material 1 [file 40529_2025_453_MOESM1_ESM.doc]

Supplementary Table 1. Primers used in this study.

| **Primers for quantitative real-time PCR analysis in *Arabidopsis* plants** | | |
| --- | --- | --- |
| **Primer names** | **Primer sequences** | **References** |
| Forward primer of *UBQ10* | 5’-CTGCGTCTTCGTGGTGGTTTCTA-3’ | Huang and Hwang 2020 |
| Reverse primer of *UBQ10* | 5’-GTCGAGTCACTTTGCAGGCGTATTA-3’ |
| Forward primer of *SAUR47*  (At3g20220) | 5’-CATCGTGCGTCTAGCAAACCAG-3’ | Wang et al. 2020 |
| Reverse primer of *SAUR47*  (At3g20220) | 5’-ATAGACCGCAAGATGTCCTCTCG-3’ |
| Forward primer of *SAUR57*  (At3g53250) | 5’-GTTGCTGCGACAAGGTTTCT-3’ | This study |
| Reverse primer of *SAUR57*  (At3g53250) | 5’-CCACTGCAACGAACAACATC-3’ |
| Forward primer of *SAUR52*  (At1g75590) | 5’-GCAGCAAGATCCGTCACATT-3’ | This study |
| Reverse primer of *SAUR52*  (At1g75590) | 5’-GATGGTTCAGATACGTCGCG-3’ |
| Forward primer of *SAUR69*  (At5g10990) | 5’-CAGGAGATTCGTGGTGCTTG-3’ | This study |
| Reverse primer of *SAUR69*  (At5g10990) | 5’-CACCGTGGCGATTCTTCTTT-3’ |
| Forward primer of *IAA15* (At1g80390) | 5’-AACGTAGATTCCTCGAGACC-3’ | Kim et al. 2022 |
| Reverse primer of *IAA15* (At1g80390) | 5’-TCTAGAGCGGTGAAAAGCTG-3’ |
| Forward primer of *GH3.1* (At2g14960) | 5’-AGGCAGCCAAGTCATGAGAC-3’ | Di et al. 2021 |
| Reverse primer of *GH3.1* (At2g14960) | 5’-TGCACCTCTTGAGATTGCGT-3’ |
| Forward primer of *XTH3*  (At3g25050) | 5’-AATTACTGCTTACGAGTTACAAGAGAAGAG-3’ | Yokoyama and Nishitani 2001 |
| Reverse primer of *XTH3*  (At3g25050) | 5’-TCTTATTTATCTTCTGCAATCATATAAAAACA-3’ |
| Forward primer of *ARR5*  (At3g48100) | 5'-AGTGCGACAAGAGCTTTACAATATCTT-3' | Higuchi et al. 2004 |
| Reverse primer of *ARR5*  (At3g48100) | 5'-CCAGGCATAGAGTAATCCGTCATT-3' |
| Forward primer of *ARR19*  (At1g49190) | 5'-TCAGCCAATTTCCGGGGAATACTAA-3' | Day et al. 2008 |
| Reverse primer of *ARR19*  (At1g49190) | 5'-TTGCAGCTTGTCAAAAACGCAAG-3' |
| Forward primer of *ARR15*  (At1g74890) | 5’-CACTCAGAGAAATCCCAGTAGTGAT-3’ | Higuchi et al. 2004 |
| Reverse primer of *ARR15*  (At1g74890) | 5’-GCAAAAACTCCTCTGCTCCTTCTAT-3’ |
| Forward primer of *GA20OX2*  (At5g51810) | 5’-TCCAACGATAATAGTGGCT-3’ | Zheng et al. 2022 |
| Reverse primer of *GA20OX2*  (At5g51810) | 5’-TTGGCATGGAGGATAATGA-3’ |
| Forward primer of *ERS2*  (At1g04310) | 5’-ACGCTTGCCAAAACATTGTA-3’ | Millenaar et al. 2005 |
| Reverse primer of *ERS2*  (At1g04310) | 5’-TGAGACGCTTTTCACCAAAC-3’ |
| Forward primer of *ERF1*  (At3g23240) | 5’-ACCGCTCCGTGAAGTTAGATAATG-3’ | An et al. 2010 |
| Reverse primer of *ERF1*  (At3g23240) | 5’-ATCCTAATCTTTCACCAAGTCCCAC-3’ |
| Forward primer of *ERF110*  (At5g50080) | 5’-ACGGTGGCGAATAAAGCAGAAGAG-3’ | Zhu et al. 2013 |
| Reverse primer of *ERF110*  (At5g50080) | 5’-GGCAGAGGTTGTTCCATTGGTGAA-3’ |
| Forward primer of *ERF14*  (At1g04370) | 5’-TCAAGGAGGTCGTAGCAGTG-3’ | Oñate-Sánchez et al. 2007 |
| Reverse primer of *ERF14*  (At1g04370) | 5’-CTCATTGAATAGGCGGCTCG-3’ |
| Forward primer of *PYL11*  (At5g45860) | 5'-ATGGAAACTTCTCAAAAATATCATACG-3' | Lim and Lee 2020 |
| Reverse primer of *PYL11*  (At5g45860) | 5'-CTCTCTCGGCTGAACTCCGCTG-3' |
| Forward primer of *ABI5*  (At2g36270) | 5'-GAGAATGCGCAGCTAAAACA-3' | Yang et al. 2023 |
| Reverse primer of *ABI5*  (At2g36270) | 5'-GTGGACAACTCGGGTTCCTC-3' |
| Forward primer of *CBF1*  (At4g25490) | 5'-TGTCTCAACTTCGCTGACTCGGC-3' | Chen et al. 2023 |
| Reverse primer of *CBF1*  (At4g25490) | 5'-ACCTTCGCTCTGTTCCGGTGTATAA-3' |
| Forward primer of *CBF2*  (At4g25470) | 5'-TGACGTGTCCTTATGGAGCTA-3' | Cho et al. 2017 |
| Reverse primer of *CBF2*  (At4g25470) | 5'-CTGCACTCAAAAACATTTGCA-3' |
| Forward primer of *AHG1*  (At5g51760) | 5'-ATCGGACGGACATGGAAGTT-3' | This study |
| Reverse primer of *AHG1*  (At5g51760) | 5'-TTCTTCTCCCTTCCACACCG-3' |
| Forward primer of *RRX33*  (At3g49110) | 5'-AATCTGTCACTTTGGCAGGAG-3' | Abuelsoud et al. 2020 |
| Reverse primer of *RRX33*  (At3g49110) | 5'-GAATGGAGCTGGAAGATTTGCG-3' |
| Forward primer of *RRX34*  (At3g49120) | 5'-CGAGAAACCATTGTAAATGAGT-3' | Kámán-Tóth et al. 2019 |
| Reverse primer of *RRX34*  (At3g49120) | 5'-CCGAGCCGAATTTGCG-3' |
| Forward primer of *MPK10*  (At3g59790) | 5'-GCGCGGATTAAAGTACATTCAT-3' | Xi et al. 2021 |
| Reverse primer of *MPK10*  (At3g59790) | 5'-GAGCCCGAAATCACAAATCTTT-3' |
| **Primers for quantitative real-time PCR analysis in pak choi plants** | | |
| **Primer names** | **Primer sequences** | **References** |
| Forward primer of *Actin-2* (*ACT1*/103869525) | 5'-CAACCAATCGTCTGTGACAA-3' | Park et al. 2014 |
| Reverse primer of *Actin-2* (*ACT1*/103869525) | 5'-ATGTCTTGGCCTACCAACAA-3' |
| Forward primer of *ARF6* | 5'-ACCTTTGTGAAGGTGTACAA-3' | This study |
| Reverse primer of *ARF6* | 5'-GCGAGCAAGCTCGCTTCGCA-3' |
| Forward primer of *ARF8* | 5'-AAGCAACCTAGCAATTACTTCTG-3' | This study |
| Reverse primer of *ARF8* | 5'-CTAGGAACAGAAAACCCTCC-3' |
| Forward primer of *ARR5* | 5'-AGCATCGTCGATCGGAAGTT-3' | This study |
| Reverse primer of *ARR5* | 5'-TTGTCTCCTACTAATCCAAG-3' |
| Forward primer of *ARR15* | 5'-CTTAGGATCTCTGCTTGTAA-3' | This study |
| Reverse primer of *ARR15* | 5'-TCACTATCAAGTTCACCTTC-3' |
| Forward primer of *ORA59* | 5'-TTCGACACTGCGGAAGAGGC-3' | This study |
| Reverse primer of *ORA59* | 5'-GCTATCACCGGAGACTCTCC-3' |

**References:**

Abuelsoud W, Cortleven A, Schmülling T (2020) Photoperiod stress induces an oxidative burst-like response and is associated with increased apoplastic peroxidase and decreased catalase activities. J Plant Physiol 253:153252.

An F, Zhao Q, Ji Y, Li W, Jiang Z, Yu X, Zhang C, Han Y, He W, Liu Y, Zhang S, Ecker JR, Guo H (2010) Ethylene-induced stabilization of ETHYLENE INSENSITIVE3 and EIN3-LIKE1 is mediated by proteasomal degradation of EIN3 binding F-box 1 and 2 that requires EIN2 in *Arabidopsis*. Plant Cell 22:2384-2401.

Chen Q, Peng L, Wang A, Yu L, Liu Y, Zhang X, Wang R, Li X, Yang Y, Li X, Wang J (2023) An R2R3-MYB FtMYB11 from tartary buckwheat has contrasting effects on abiotic tolerance in *Arabidopsis*. J Plant Physiol 280:153842.

Cho S, Yu SL, Park J (2017) Accession-dependent CBF gene deletion by CRISPR/Cas system in *Arabidopsis*. Front Plant Sci 8:1910.

Day RC, Herridge RP, Ambrose BA, Macknight RC (2008) Transcriptome analysis of proliferating *Arabidopsis* endosperm reveals biological implications for the control of syncytial division, cytokinin signaling, and gene expression regulation. Plant Physiol 148:1964-1984.

Di DW, Sun L, Wang M, Wu J, Kronzucker HJ, Fang S, Chu J, Shi W, Li G (2021) WRKY46 promotes ammonium tolerance in *Arabidopsis* by repressing NUDX9 and indole-3-acetic acid-conjugating genes and by inhibiting ammonium efflux in the root elongation zone. New Phytol 232:190-207.

Higuchi M, Pischke MS, Mähönen AP, Miyawaki K, Hashimoto Y, Seki M, Kobayashi M, Shinozaki K, Kato T, Tabata S, Helariutta Y, Sussman MR, Kakimoto T (2004) In planta functions of the *Arabidopsis* cytokinin receptor family. Proc Natl Acad Sci USA 101:8821-8826.

Huang FC, Hwang HH (2020) *Arabidopsis* RETICULON-LIKE4 (RTNLB4) protein participates in *Agrobacterium* infection and VirB2 peptide-induced plant defense response. Int J Mol Sci 21:1722.

Kámán-Tóth E, Dankó T, Gullner G, Bozsó Z, Palkovics L, Pogány M (2019) Contribution of cell wall peroxidase- and NADPH oxidase-derived reactive oxygen species to *Alternaria brassicicola*-induced oxidative burst in *Arabidopsis*. Mol Plant Pathol 20:485-499.

Kim SH, Bahk S, Nguyen NT, Pham MLA, Kadam US, Hong JC, Chung WS (2022) Phosphorylation of the auxin signaling transcriptional repressor IAA15 by MPKs is required for the suppression of root development under drought stress in *Arabidopsis*. Nucleic Acids Res 50:10544-10561.

Lim CW, Lee SC (2020) ABA-dependent and ABA-independent functions of RCAR5/PYL11 in response to cold stress. Front Plant Sci 11:587620.

Millenaar FF, Cox MC, van Berkel YEdJ, Welschen RA, Pierik R, Voesenek LA, Peeters AJ (2005) Ethylene-induced differential growth of petioles in *Arabidopsis*. Analyzing natural variation, response kinetics, and regulation. Plant Physiol 137:998-1008.

Oñate-Sánchez L, Anderson JP, Young J, Singh KB (2007) AtERF14, a member of the ERF family of transcription factors, plays a nonredundant role in plant defense. Plant Physiol 143:400-409.

Park JI, Ahmed NU, Jung HJ, Arasan SK, Chung MY, Cho YG, Watanabe M, Nou IS (2014) Identification and characterization of LIM gene family in *Brassica rapa*. BMC Genomics 15:641.

Wang J, Sun N, Zhang F, Yu R, Chen H, Deng XW, Wei N (2020) SAUR17 and SAUR50 differentially regulate PP2C-D1 during apical hook development and cotyledon opening in *Arabidopsis*. Plant Cell 32:3792-3811.

Xi X, Hu Z, Nie X, Meng M, Xu H, Li J (2021) Cross inhibition of MPK10 and WRKY10 participating in the growth of endosperm in *Arabidopsis thaliana*. Front Plant Sci 12:640346.

Yang J, He R, Qu Z, Gu J, Jiang L, Zhan X, Gao Y, Adelson DL, Li S, Wang ZY, Zhu Y, Wang D (2023) Long noncoding RNA ARTA controls ABA response through MYB7 nuclear trafficking in *Arabidopsis.* Dev Cell 58:1206-1217.

Yokoyama R, Nishitani K (2001) A comprehensive expression analysis of all members of a gene family encoding cell-wall enzymes allowed us to predict cis-regulatory regions involved in cell-wall construction in specific organs of *Arabidopsis.* Plant Cell Physiol 42:1025-1033.

Zheng F, Wang Y, Gu D, Liu X (2022) Histone deacetylase HDA15 restrains PHYB-dependent seed germination via directly repressing GA20ox1/2 gene expression. Cells 11:3788.

Zhu L, Liu D, Li Y, Li N (2013) Functional phosphoproteomic analysis reveals that a serine-62-phosphorylated isoform of ethylene response factor110 is involved in *Arabidopsis* bolting. Plant Physiol 161:904-917.


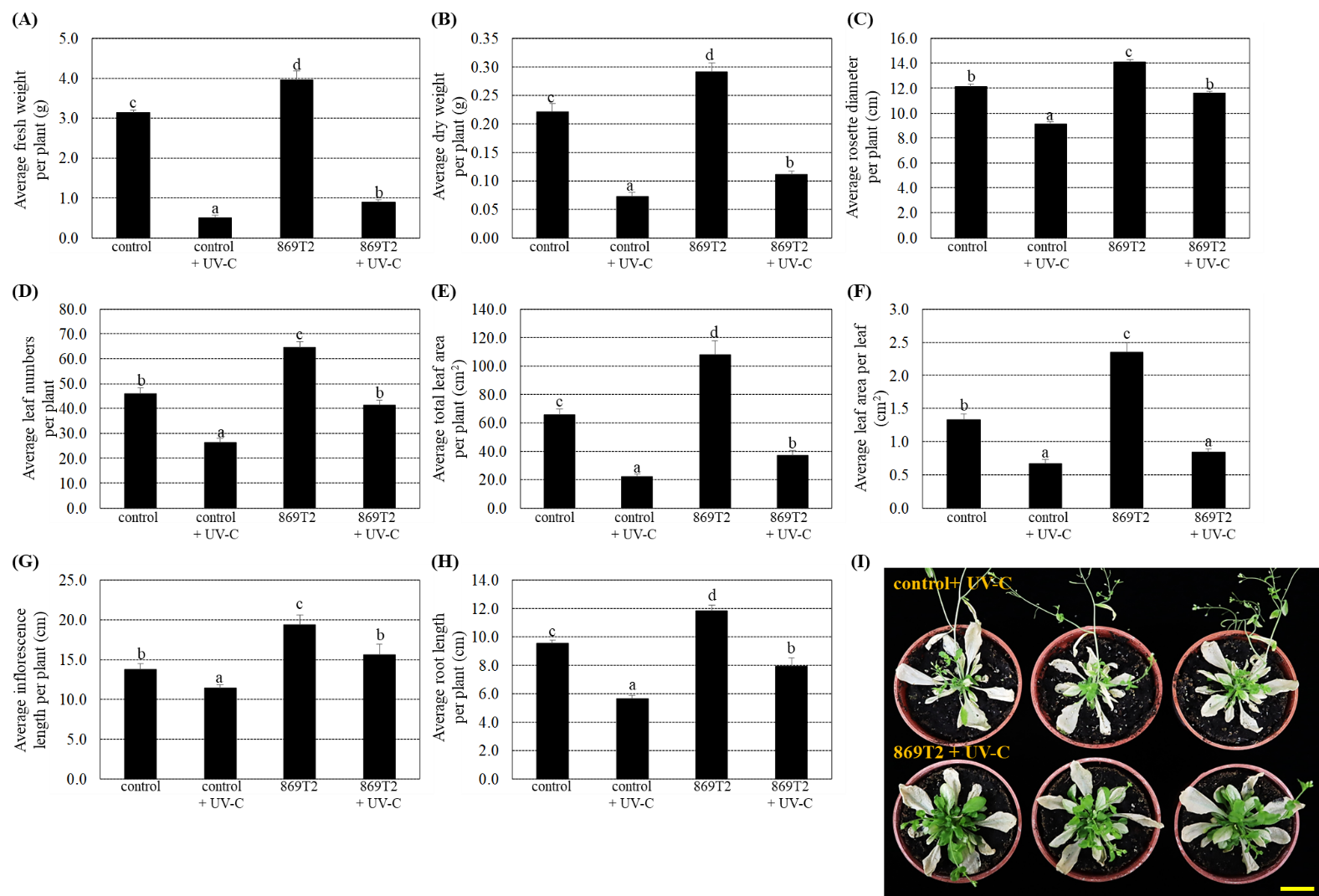


**Supplementary Figure. S1.** Under UV-C stress treatments, inoculations of the strain 869T2 in the *Arabidopsis* increased its growth compared to the mock-inoculated control plants. After 869T2 inoculations, the plants were exposed to Sankyo Denki 20-w UV-C lamps (253 nm) for 40 minutes and subsequently recovered for seven days under normal light conditions. After UV-C stress treatments, the average fresh weight per plant (Panel A), the average dry weight per plant (Panel B), the average rosette diameter per plant (Panel C), the average leaf numbers per plant (Panel D), the average total leaf area per plant (Panel E), the average leaf area per leaf (Panel F), the average inflorescence length per plant (Panel G), the average root length per plant (Panel H), and the top-view (Panel I) photographs of the control and the 869T2-inoculated plants under UV-C stress and non-stress conditions were recorded. Data are mean±SE (standard error) from at least three independent bacteria inoculation experiments. More than 20 individual plants were examined for each bacteria inoculation assay. Data were analyzed by Duncan tests and means with different letters were significantly different (*p* < 0.05). Yellow bar = 3 cm.


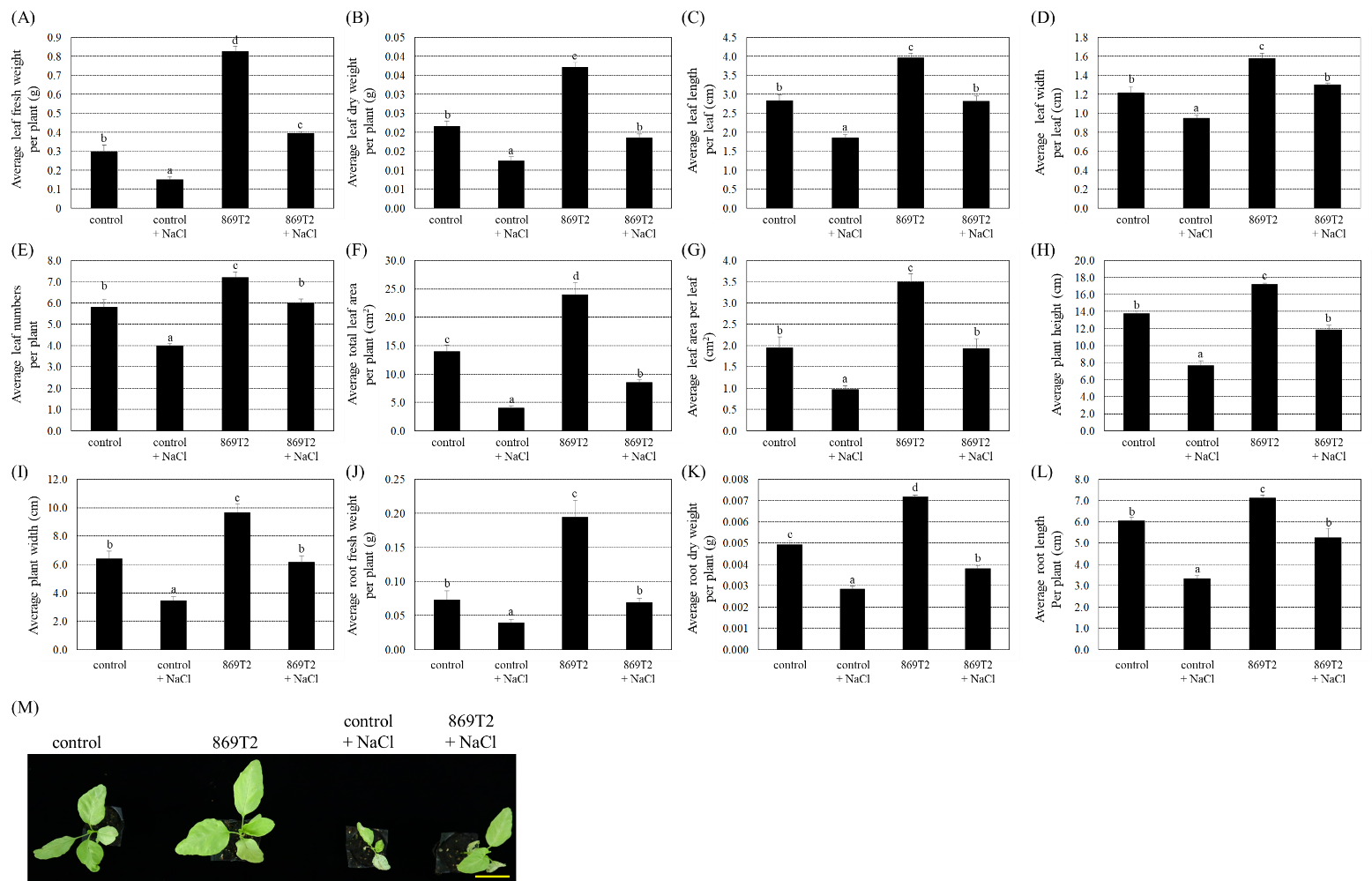


**Supplementary Figure. S2.** Under salt stress conditions, inoculation with the strain 869T2 significantly increased various growth parameters of the Chinese Amaranth (*Amaranthus tricolor*) plants compared to the mock-inoculated control plants. Following inoculation, the plants were subjected to salt stress by irrigation with the 250 mM NaCl solutions for seven days and subsequently allowed to recover for three days by re-watering with distilled water. Post-treatment, the following growth parameters were measured and analyzed: average leaf fresh weight per plant (Panel A), average leaf dry weight per plant (Panel B), average leaf length per leaf (Panel C), average leaf width per leaf (Panel D), average leaf numbers per plant (Panel E), average total leaf area per plant (Panel F), average leaf area per leaf (Panel G), average plant height (Panel H), average plant width (Panel I), average root fresh weight per plant (Panel J), average root dry weight per plant (Panel K), and average root length per plant (Panel L). Additionally, top-view photographs of the mock-inoculated and 869T2-inoculated plants under both salt stress and non-stress conditions were taken for visual comparison (Panel M). Data represent the mean ± standard error (SE) derived from at least three independent bacterial inoculation experiments, with more than 20 individual plants examined per treatment. Statistical analysis was conducted using Duncan test, and means marked with different letters were considered significantly different at *p* < 0.05. Yellow bar = 3 cm.


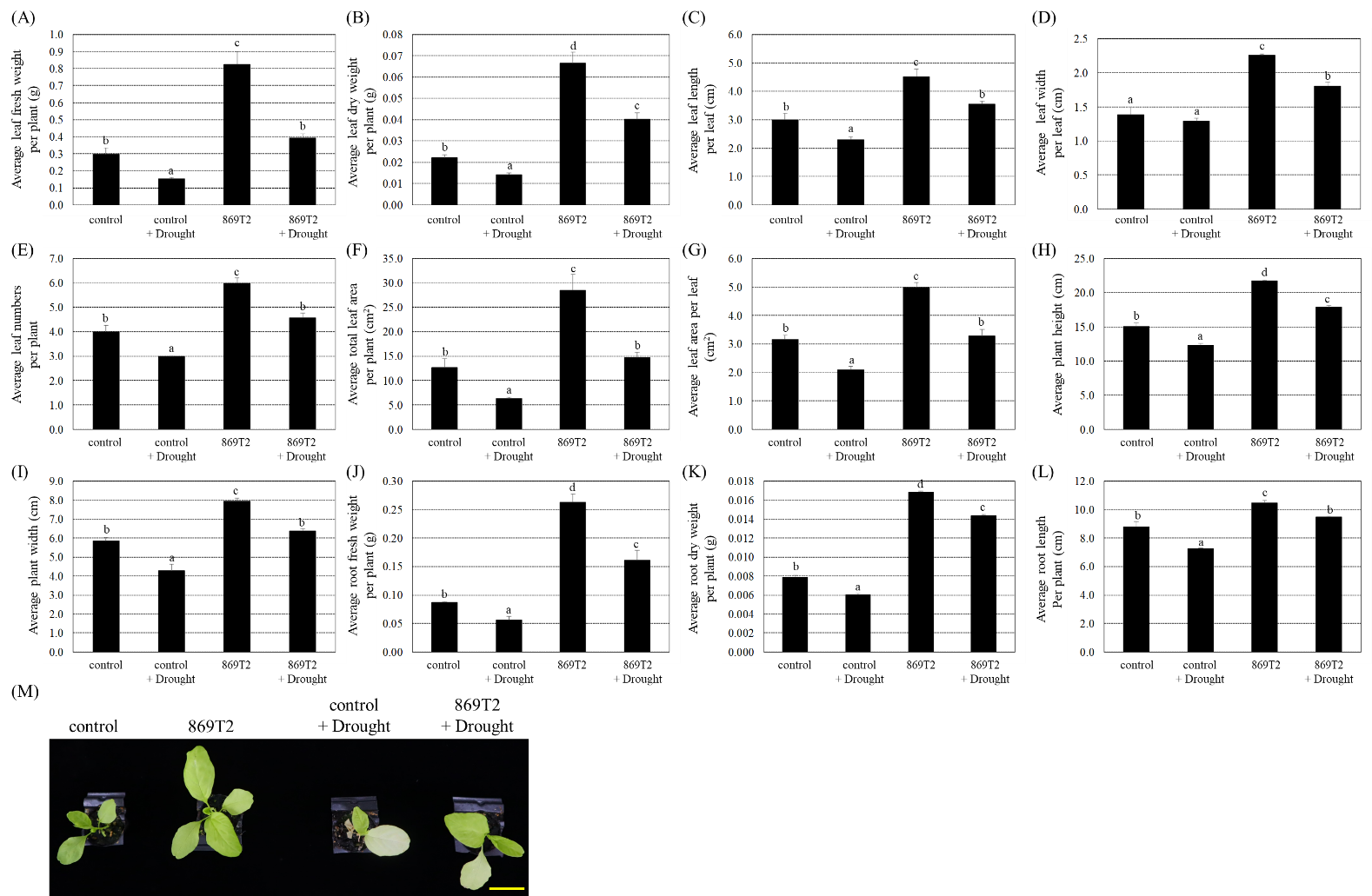


**Supplementary Figure. S3.** Under drought stress conditions, inoculation with the strain 869T2 significantly improved the growth and biomass accumulation of Chinese amaranth (*Amaranthus tricolor*) compared to mock-inoculated controls. After bacterial inoculation, plants were subjected to drought stress by withholding water for seven days, followed by a three-day recovery period through re-watering with distilled water. Post-treatment, the following growth parameters were evaluated: average leaf fresh weight per plant (Panel A), average leaf dry weight per plant (Panel B), average leaf length per leaf (Panel C), average leaf width per leaf (Panel D), average number of leaves per plant (Panel E), total leaf area per plant (Panel F), average leaf area per leaf (Panel G), average plant height (Panel H), average plant width (Panel I), average root fresh weight per plant (Panel J), average root dry weight per plant (Panel K), and average root length per plant (Panel L). Top-view photographs of mock-inoculated and 869T2-inoculated plants under both drought stress and non-stress conditions were also captured for visual assessment (Panel M). Data are presented as mean ± standard error (SE), based on at least three independent bacterial inoculation experiments involving more than 20 individual plants per treatment. Statistical significance was determined using Duncan test, with means labeled by different letters considered significantly different at *p* < 0.05. Yellow bar = 3 cm.
